# Identification of risk loci for postpartum depression in a genome‐wide association study

**DOI:** 10.1111/pcn.13731

**Published:** 2024-09-17

**Authors:** Xue Li, Nagahide Takahashi, Akira Narita, Yukako Nakamura, Mika Sakurai‐Yageta, Keiko Murakami, Mami Ishikuro, Taku Obara, Masahiro Kikuya, Fumihiko Ueno, Hirohito Metoki, Hisashi Ohseto, Ippei Takahashi, Tomohiro Nakamura, Noriko Warita, Tomoka Shoji, Zhiqian Yu, Chiaki Ono, Natsuko Kobayashi, Saya Kikuchi, Tasuku Matsuki, Fuji Nagami, Soichi Ogishima, Junichi Sugawara, Tetsuro Hoshiai, Masatoshi Saito, Nobuo Fuse, Kengo Kinoshita, Masayuki Yamamoto, Nobuo Yaegashi, Norio Ozaki, Gen Tamiya, Shinichi Kuriyama, Hiroaki Tomita

**Affiliations:** ^1^ Department of Psychiatry Tohoku University Graduate School of Medicine Sendai Japan; ^2^ Department of Regional Alliance for Promoting Liaison Psychiatry Tohoku University Graduate School of Medicine Sendai Japan; ^3^ Department of Child and Adolescent Psychiatry Nagoya University Graduate School of Medicine Nagoya Japan; ^4^ Department of Integrative Genomics Tohoku University Tohoku Medical Megabank Organization Sendai Japan; ^5^ Department of Psychiatry Nagoya University Graduate School of Medicine Nagoya Japan; ^6^ Department of Preventive Medicine and Epidemiology Tohoku University Tohoku Medical Megabank Organization Sendai Japan; ^7^ Department of Hygiene and Public Health Teikyo University School of Medicine Tokyo Japan; ^8^ Department of Health Record Informatics Tohoku University Tohoku Medical Megabank Organization Sendai Japan; ^9^ Department of Psychiatry Tohoku University Hospital Sendai Japan; ^10^ Department of Public Relations and Planning Tohoku University Tohoku Medical Megabank Organization Sendai Japan; ^11^ Department of Community Medical Supports Tohoku University Tohoku Medical Megabank Organization Sendai Japan; ^12^ Department of gynecology and obstetrics Tohoku University Graduate School of Medicine Sendai Japan; ^13^ Suzuki Memorial Hospital Iwanumashi Japan; ^14^ Pathophysiology of Mental Disorders Nagoya University Graduate School of Medicine Nagoya Japan; ^15^ Tohoku University International Research Institute of Disaster Sciences Sendai Japan

**Keywords:** genome‐wide association study, meta‐analysis, pathway analyses, perinatal women, postpartum depression

## Abstract

**Aim:**

Genome‐wide association studies (GWAS) of postpartum depression (PPD) based on accumulated cohorts with multiple ethnic backgrounds have failed to identify significantly associated loci. Herein, we conducted a GWAS of Japanese perinatal women along with detailed confounding information to uncover PPD‐associated loci.

**Methods:**

The first and second cohorts (*n* = 9260 and *n* = 8582 perinatal women enrolled in the Tohoku Medical Megabank Project) and the third cohort (*n* = 997), recruited at Nagoya University, underwent genotyping. Of them, 1421, 1264, and 225 were classified as PPD based on the Edinburgh Postnatal Depression Scale 1 month after delivery. The most influential confounding factors of genetic liability to PPD were selected, and logistic regression analyses were performed to evaluate genetic associations with PPD after adjusting for confounders.

**Results:**

A meta‐analysis of GWAS results from the three cohorts identified significant associations between PPD and the following loci (*P* < 5 × 10^−8^) by integrating the number of deliveries and the number of family members living together as the most influential confounders: rs377546683 at *DAB1*, rs11940752 near *UGT8*, rs141172317, rs117928019, rs76631412, rs118131805 at *DOCK2*, rs188907279 near *ZNF572*, rs504378, rs690150, rs491868, rs689917, rs474978, rs690118, rs690253 near *DIRAS2*, rs1435984417 at *ZNF618*, rs57705782 near *PTPRM*, and rs185293917 near *PDGFB*. Pathway analyses indicated that SNPs suggestively associated with PPD were mostly over‐represented in categories including long‐term depression, GnRH signaling, glutamatergic synapse, oxytocin signaling, and Rap1 signaling.

**Conclusion:**

The current GWAS study identified eight loci significantly associated with PPD, which may clarify the genetic structure underlying its pathogenesis.

Postpartum depression (PPD) refers to a depressive state occurring after birth, typically within the first month after delivery.^1^, ^2^ The World Health Organization has reported that 10–20% of perinatal women experience depressive symptoms during the postpartum period.^3^ Despite the high prevalence and negative consequences of PPD, little is known about its underlying biology. For example, research has shown evidence to support the links between PPD and dysfunctions in hormones,^4^, ^5^ including estrogen,^6^, ^7^, ^8^ progesterone,^6^, ^9^ and oxytocin,^10^, ^11^ inflammatory systems,^12^ plasma metabolic^13^ and neurotransmission systems including GABA^14^, ^15^, ^16^ and glutamate signaling.^17^, ^18^

Candidate gene approaches to PPD targeting genes implicated in neurotransmitter synthesis, metabolism, and neural growth have yielded mixed results. For instance, whereas Comasco *et al*.^19^ found a significant association between *BDNF* and PPD onset in 275 women, Figueira *et al*.^20^ observed no significant differences in *BDNF* genotypes in 227 participants. Similarly, while Fasching^21^ reported significant associations between TPH2 and PPD in a Caucasian cohort, Khabour *et al*.^22^ detected no linkage in 370 Jordanians. The variable outcomes and small sample sizes limit the definitive determination of genetic susceptibility, overshadowed by the disorder's complexity and the interaction between genetic predispositions and environmental factors.

Recent genome‐wide association studies (GWAS) have enriched our understanding of the genetic foundations of depression and major depressive disorder (MDD).^23^, ^24^ These studies, including those by Meng *et al*., Wray *et al*., Giannakopoulou *et al*., and Howard^25^, ^26^, ^27^, ^28^ have collectively illuminated the complex genetic architecture of depression, identifying a diverse array of risk loci and genetic variants that are crucial for comprehending this disorder. Despite numerous GWAS studies on PPD,^29^, ^30^ definitive genetic variants associated with the condition remain elusive. Guintivano *et al*.^30^ conducted an extensive GWAS encompassing 18,770 women diagnosed with PPD and 58,461 controls across 18 European, one East Asian, and one African ancestry cohorts. The comprehensive meta‐analyses and post‐GWAS assessments, including single nucleotide polymorphism (SNP)‐based heritability, genetic correlations with other psychiatric disorders, and targeted enrichment analyses in specific tissues and cell types, failed to identify any SNPs reaching genome‐wide significance. Notably, despite PPD's expected homogeneity as a subtype of MDD in females of reproductive age affected by a joint biopsychosocial event and its higher twin heritability at 54% compared to MDD's 32%,^31^ the genetic underpinnings of PPD remain undefined.

Thus, a GWAS of Japanese perinatal women, along with detailed confounding information, was performed to uncover PPD‐associated loci.

## Materials and Methods

### Study design and participants

This study drew on Tohoku Medical Megabank Organization (ToMMo) and Nagoya University cohort data. The ToMMo initiated the TMM BirThree Cohort Study,^32^, ^33^ enrolling three generations of families, including newborns, their siblings, parents, and grandparents from Miyagi Prefecture between 2014 and 2018. Participating mothers recruited during pregnancy had their mental health assessed both pre‐ and post‐delivery. Subjects from the initial phase (*n* = 9260) and subsequent phase (*n* = 8582) underwent whole‐genome genotyping using the Japonica Array version 2^34^ and NEO,^35^ designated the TMM‐V2 and TMM‐NEO cohorts, respectively. In addition, a cohort of 997 perinatal women from Aichi Prefecture, recruited by Nagoya University, was genotyped with Japonica Array NEO, referred to as the NGO‐NEO cohort. All participants have given written informed consent before participation, and their anonymity has been preserved. The protocols of the present study were approved by the Ethics Committees of Tohoku University Graduate School of Medicine (Certification #: 2021‐4‐137 and 2021‐1‐266) and Nagoya University Graduate School of Medicine (Certification #: 2007‐0513). The protocols complied with the relevant laws and ethical guidelines issued by the Japanese government, relevant academic associations, and the Declaration of Helsinki.

### Genotyping, quality control, and imputation

To exclude individual‐level rare variants, criteria were applied, including individual missingness (F_MISS >0.05) and heterozygosity and inbreeding (*F*‐value >0.2 and < −0.2) using PLINK 1.9.^36^ For single nucleotide polymorphism (SNP)‐level rare variants, thresholds for SNP missingness (F_MISS >0.05), deviations from Hardy–Weinberg Equilibrium (range from 0.001 to 0.000001), and minor allele frequency (MAF <0.01) were set. Following these quality control measures, the remaining SNPs underwent GWAS analysis. The genotype data were pre‐phased using SHAPEIT2^37^ with the–duohmm^38^ option to enhance phasing accuracy by incorporating relatedness information between individuals. Subsequently, genotypes were imputed using IMPUTE2 against the 3.5KJPNv2 reference panel of haplotypes.^39^ The imputation process involved masking input SNPs and comparing the imputed SNPs to the masked ones to calculate *r* ^2^, assessing the precision across MAF bins. In addition, the information measure (INFO score) from IMPUTE2, ranging from 0 to 1, was used to evaluate imputation quality for each marker.^40^, ^41^

### Measures

In the present study, PPD was evaluated using the Edinburgh Postnatal Depression Scale (EPDS).^42^ The cases (i.e., PPD) and controls were defined based on EPDS scores (≥9 and <9, respectively) because many studies conducted in Japan have used this value as the cutoff score for PPD requiring intervention in Japanese perinatal women.^43^, ^44^ Of the total subjects of TMM‐V2, TMM‐NEO, and NGO‐NEO cohorts, 1421 (15.3%), 1264 (14.7%), and 225 (22.6%) subjects were classified as PPD, respectively. Seven confounding factors were estimated based on previous studies to indicate associations with PPD^45^, ^46^, ^47^, ^48^: marital status, cohabitation status with a partner, number of deliveries, income, education history, number of family members living together, and the method of conception. Detailed demographic and psychosocial data regarding the confounding factors were linked for 6191, 5353, and 649 participants in the TMM‐V2, TMM‐NEO, and NGO‐NEO cohorts, among which 908 (14.7%), 741 (13.8%), and 111 (17.1%) were classified as PPD, respectively.

### Statistical analysis

The genotype data from the three cohorts underwent imputation and quality control before GWAS analysis was performed, using age and population stratification from principal component analysis (PCA) as confounding factors. Further, a meta‐analysis, as detailed in Supplementary Methods of the Supplementary Material was conducted. To address potential biases due to blood relationships and unbalanced case–control group sizes in the GWAS results for PPD, GCTA fastGWA and REGENIE, which consider these factors, were used.^36^, ^49^, ^50^, ^51^, ^52^, ^53^, ^54^ After using machine learning techniques to prioritize confounding factors based on their impact on PPD, GWAS was performed using data from subjects with complete information on these prioritized factors. Furthermore, restricted maximum likelihood analysis (REML) was used to estimate the effects of all SNPs. Finally, pathway analysis was applied to identify biological pathways implicated in PPD, as defined by the Kyoto Encyclopedia of Genes and Genomes (KEGG) functional database^55^, ^56^, ^57^, ^58^, ^59^ (refer to Text S2 in the Supplementary Material).

## Results

### Genome‐wide association with the PPD phenotype in the TMM‐V2, TMM‐NEO, and NGO‐NEO cohorts considering PCA and age as confounding factors

There were no loci associated with PPD at a genome‐wide significance level in the TMM‐V2, TMM‐NEO, and NGO‐NEO cohorts. There were 75, 68, and 12 PPD‐associated loci with a suggestive significance threshold of 10^−5^ (Figs S1–S3).

### Meta‐analysis of PPD GWAS of the TMM‐V2, TMM‐NEO, and NGO‐NEO cohorts considering PCA and age as confounding factors

The meta‐analysis of the three cohorts (TMM‐V2, TMM‐NEO, and NGO‐NEO) revealed that 571 loci were associated with PPD with a suggestive significance threshold of 10^−5^; however, no loci reached a genome‐wide significance level (Fig. S4). The meta‐analysis of the same data set based on GCTA fastGWA and REGENIE, controlling for potential genetic relationships among the participants and an unbalanced number of participants between cases and control, replicated no loci associated with PPD at a genome‐wide significance level (Fig. S5).

### Genome‐wide association with the PPD phenotype in the TMM‐V2, TMM‐NEO, and NGO‐NEO cohorts considering the multiple potential confounding factors

Since previous studies have reported that various factors, including marital status, cohabitation status with a partner, number of deliveries, income, education history, number of family members living together, and the method of conception, are largely associated with PPD, these potential major confounding factors were integrated into the GWAS analysis. Considering these confounding factors, the GWAS analysis of TMM‐V2 indicated that two genomic regions, located at 8q24.13 (rs188907279, *P* = 3.58022E‐08) near *ZNF572* and 18p11.23 (rs57705782, *P* = 2.8088E‐08) near *PTPRM*, were loci associated with PPD above the whole genome‐wide significance threshold of 5 × 10^−8^. The association results for significant SNPs in TMM‐V2 with all confounding factors are shown in Table 1, and a Manhattan plot of the results is shown in Fig. S6.

**Table 1:** SNPs significantly associated with postpartum depression in the TMM‐V2 and TMM‐NEO cohorts considering the multiple potential confounding factors

Cohort	SNP ID	BP	Chr#	Locus	Gene	EA/RA	EAF	*P*‐value	OR (95% CI)	LOG(OR)_SE	SE
TMM‐V2	rs188907279	125,991,737	8	q24.13	*ZNF572*	A/T	0.02	3.58E‐08	1.54 (0.72–3.31)	0.17	0.39
	rs57705782	7,451,115	18	p11.23	*PTPRM*	T/C	0.80	2.81E‐08	1.16 (0.45–2.97)	0.07	0.48
TMM‐NEO	rs377546683	58,800,275	1	p32.2	*DAB1*	C/G	0.06	3.85E‐08	1.34 (0.36–4.99)	0.20	0.67
	rs138021793	150,619,600	3	q25.1	*RP11‐166N6*.*3*	T/G	0.02	1.60E‐08	2.78 (1.95–3.95)	0.18	0.18
	rs11940752	115,442,324	4	q26	*UGT8*	A/T	0.96	2.95E‐08	2.13 (1.50–3.03)	0.14	0.18
	rs117928019	169,226,605	5	q35.1	*DOCK2*	C/G	0.02	2.05E‐08	2.62 (1.84–3.73)	0.17	0.18
	rs185293917	39,604,656	22	q13.1	*PDGFB*	G/C	0.01	9.58E‐09	3.25 (2.33–4.54)	0.21	0.17

The single nucleotide polymorphisms (SNPs) listed in the table were significantly associated with postpartum depression in the Tohoku Medical Megabank Organization perinatal women sub‐cohort genotyped by the Japonica Array version 2 (TMM‐V2) and the Tohoku Medical Megabank Organization perinatal women sub‐cohort genotyped by the Japonica Array version NEO (TMM‐NEO) considering the multiple potential confounding factors. The Q statistics and *I* ^2^ values for all SNPs were zero in each TMM‐V2 and TMM‐NEO cohort, indicating no heterogeneity within the cohorts.

BP, base pair position; Chr #, chromosome number; EA, effect allele; RA, reference allele; EAF, effect allele frequency; OR (95% CI), odds ratio (95% confidence interval); LOG(OR)_SE, standard error of the log odds ratio; SE, standard error.

The GWAS analysis of TMM‐NEO considering the confounding factors indicated that five genomic regions, located at 1p32.2 (rs377546683, *P* = 3.85122E‐08) in the *DAB1* region, 3q25.1 (rs138021793, *P* = 1.59838E‐08) and 4q26 (rs115442324, *P* = 2.9459E‐08) near *UGT8*, 5q35.1 (rs117928019, *P* = 2.05066E‐08) in the *DOCK2* region, and 22q13.1 (rs185293917, *P* = 9.57557E‐09) near *PDGFB*, were loci associated with PPD above the whole genome‐wide significance threshold of 5 × 10–8. The association results for significant SNPs in TMM‐V2 with all the confounding factors are shown in Table 1, and a Manhattan plot of the results is shown in Fig. S7.

The GWAS analysis of NGO‐NEO considering the confounding factors indicated no genomic regions associated with PPD above the whole genome‐wide significance threshold of 5 × 10^−8^ (Table S1, Fig. S8).

### Meta‐analysis of PPD GWAS of TMM‐V2, TMM‐NEO, and NGO‐NEO cohorts considering the multiple potential confounding factors

A meta‐analysis integrating full GWAS results from three cohorts and considering major confounding factors confirmed significant associations at six loci, except for rs138021793 on 3q25.1. It also identified additional PPD‐associated loci near the *DIRAS2* gene region on 9q22.2, including rs504378 (*P* = 2.022E‐09), rs690150 (*P* = 5.158E‐09), rs491868 (*P* = 5.831E‐09), rs689917 (*P* = 7.838E‐09), rs474978 (*P* = 5.769E‐09), rs690118 (*P* = 2.597E‐08) and rs690253 (*P* = 3.250E‐09), as well as near the *ZNF618* gene region on 9q31.3 (rs1435984417, *P* = 3.555E‐08), all surpassing the genome‐wide significance threshold (*P* < 5 × 10–8). These associations and confounding factors are detailed in Table 2 and Table S3, with a Manhattan plot provided in Fig. S9. Validation analyses using GCTA fastGWA and REGENIE, which adjusted for potential genetic relationships and imbalances between cases and controls, successfully replicated all identified loci linked to PPD at a genome‐wide significance level, as illustrated in Fig. S10.

**Table 2:** SNPs significantly associated with postpartum depression in the meta‐analysis of TMM‐V2, TMM‐NEO, and NGO‐NEO cohorts considering the multiple potential confounding factors

SNP ID	BP	Chr #	Gene	Locus	EAF	*P*‐value
rs377546683	58,800,275	1	*DAB1*	p32.2	0.06	3.62E‐09
rs11940752	115,442,324	4	*UGT8*	q26	0.96	8.73E‐09
rs117928019	169,226,605	5	*DOCK2*	q35.1	0.02	4.61E‐08
rs188907279	125,991,737	8	*ZNF572*	q24.13	0.02	3.13E‐08
rs504378	93,336,214	9	*DIRAS2*	q22.2	0.10	2.02E‐09
rs690150	93,359,638	9	*DIRAS2*	q22.2	0.10	5.16E‐09
rs491868	93,349,476	9	*DIRAS2*	q22.2	0.10	5.83E‐09
rs689917	93,359,512	9	*DIRAS2*	q22.2	0.10	7.84E‐09
rs474978	93,352,826	9	*DIRAS2*	q22.2	0.10	5.77E‐09
rs690118	93,356,340	9	*DIRAS2*	q22.2	0.11	2.60E‐08
rs690253	93,352,854	9	*DIRAS2*	q22.2	0.10	3.25E‐09
rs1435984417	113,929,504	9	*ZNF618*	q31.3	0.02	3.56E‐08
rs57705782	7,451,115	18	*PTPRM*	p11.23	0.80	1.86E‐09
rs185293917	39,604,656	22	*PDGFB*	q13.1	0.01	2.33E‐08

The single nucleotide polymorphisms (SNPs) listed in the table were significantly associated with postpartum depression in the meta‐analysis of Tohoku Medical Megabank Organization perinatal women sub‐cohort genotyped by the Japonica Array version 2 (TMM‐V2), Tohoku Medical Megabank Organization perinatal women sub‐cohort genotyped by the Japonica Array version NEO (TMM‐NEO), and Nagoya University perinatal women cohort genotyped by the Japonica Array version NEO (NGO‐NEO) considering the multiple potential confounding factors.

BP, base pair position; Chr #, chromosome number; EAF, effect allele frequency.

### Evaluations of each feature

**Fig. 1:**
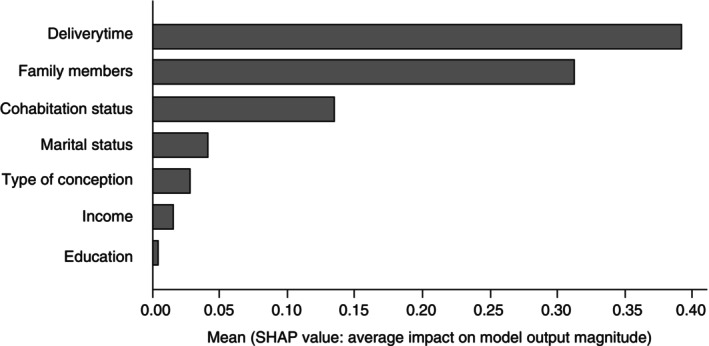
The importance scores of factors in predicting postpartum depression. The figure shows the importance scores of each confounding factor based on the feature attribution analysis using the SHapley Additive exPlanations approach to reveal specific confounding factors of the whole genome association study of postpartum depression.

A feature attribution analysis using the SHapley Additive exPlanations (SHAP) approach was performed to reveal confounding factors. The importance scores of each confounding factor showed that, compared with the other five factors, both the number of deliveries and the number of family members living together were highly related to EPDS. The importance scores of each confounding factor are plotted in Fig. 1.

### Genome‐wide association with the PPD phenotype in the TMM‐V2, TMM‐NEO, and NGO‐NEO cohorts considering the two most important confounding factors

Genome‐wide significance was obtained in two genomic regions, located at 8q24.13 (rs188907279, *P* = 4.58287E‐08) near the *ZNF572* gene region and 18p11.23 (rs57705782, *P* = 2.810E‐08) near the *PTPRM* gene region, with *P*‐values above the whole genome‐wide significance threshold of 5 × 10^−8^. The association results for significant SNPs in TMM‐V2 with the important confounding factors are shown in Table 3, and a Manhattan plot of the results is shown in Fig. S11.

**Table 3:** SNPs significantly associated with postpartum depression in the TMM‐V2 and TMM‐NEO cohorts considering the two most important confounding factors

Cohort	SNP ID	BP	Chr#	Locus	Gene	EA/RA	EAF	*P*‐value	OR (95% CI)	LOG(OR)_SE	SE
TMM‐V2	rs188907279	125,991,737	8	q24.13	*ZNF572*	A/T	0.02	4.58E‐08	1.47 (0.61–3.55)	0.17	0.45
	rs57705782	7,451,115	18	p11.23	*PTPRM*	T/C	0.80	3.98E‐08	1.19 (0.12–11.79)	0.20	1.17
TMM‐NEO	rs377546683	58,800,275	1	p32.2	*DAB1*	C/G	0.02	3.85E‐08	1.34 (0.35–5.07)	0.20	0.68
	rs138021793	150,619,600	3	q25.1	*RP11‐166N6*.*3*	T/G	0.02	2.84E‐08	2.71 (1.90–3.86)	0.18	0.18
	rs11940752	115,442,324	4	q26	*UGT8*	A/T	0.96	2.48E‐08	2.12 (1.49–3.02)	0.14	0.18
	rs141172317	169,216,010	5	q35.1	*DOCK2*	T/C	0.02	4.61E‐08	2.78 (1.95–3.98)	0.19	0.18
	rs117928019	169,226,605	5	q35.1	*DOCK2*	C/G	0.02	6.51E‐09	2.69 (1.91–3.76)	0.17	0.17
	rs76631412	169,259,673	5	q35.1	*DOCK2*	A/T	0.02	3.36E‐08	2.71 (1.90–3.87)	0.18	0.18
	rs118131805	169,267,720	5	q35.1	*DOCK2*	T/G	0.02	3.36E‐08	2.71 (1.90–3.87)	0.18	0.18
	rs185293917	39,604,656	22	q13.1	*PDGFB*	G/C	0.01	1.52E‐08	3.15 (2.23–4.46)	0.20	0.18

The single nucleotide polymorphisms (SNPs) listed in the table were significantly associated with postpartum depression in the Tohoku Medical Megabank Organization perinatal women sub‐cohort genotyped by the Japonica Array version 2 (TMM‐V2) and the Tohoku Medical Megabank Organization perinatal women sub‐cohort genotyped by the Japonica Array version NEO (TMM‐NEO) considering the two most important confounding factors. The Q statistics and *I* ^2^ values for all SNPs were zero in each TMM‐V2 and TMM‐NEO cohort, indicating no heterogeneity within the cohorts.

BP, base pair position; Chr #, chromosome number; EA, effect allele; RA, reference allele; EAF, effect allele frequency; OR (95% CI), odds ratio (95% confidence interval); LOG(OR)_SE, standard error of the log odds ratio; SE, standard error.

Genome‐wide significance was obtained for five genomic regions, located at 1p32.2 (rs377546683, *P* = 3.851E‐08) in the DAB1 gene region, 3q25.1 (rs138021793, *P* = 2.841E‐08) and 4q26 (rs115442324, *P* = 2.481E‐08) near the UGT8 gene region, 5q35.1 (rs117928019, *P* = 6.513E‐09), 5q35.1 (rs141172317, *P* = 4·613E‐08), 5q35.1 (rs76631412, *P* = 3.363E‐08), and 5q35.1 (rs118131805, *P* = 3.363E‐08) in the *DOCK2* gene region, and 22q13.1 (rs185293917, *P* = 1.519E‐08) near the *PDGFB* gene region, with *P*‐values above the whole genome‐wide significance threshold of 5 × 10^−8^. The association results for significant SNPs in TMM‐NEO with the important confounding factors are shown in Table 3, and a Manhattan plot of the results is shown in Fig. S12.

The GWAS analysis of NGO‐NEO, considering the two important confounding factors, indicated no genomic regions associated with PPD above the whole genome‐wide significance threshold of 5 × 10^−8^ (Table S2, Fig. S13).

### Meta‐analysis of PPD GWAS of TMM‐V2, TMM‐NEO, and NGO‐NEO cohorts considering the two most important confounding factors

A meta‐analysis of complete GWAS data from three cohorts, accounting for major confounders, confirmed significant associations at nine loci, except for rs138021793 on 3q25.1. This analysis identified additional loci associated with PPD near the *DIRAS2* gene region on 9q22.2, including rs504378 (*P* = 4.119E‐09), rs690150 (*P* = 2.835E‐09), rs491868 (*P* = 4.237E‐09), rs689917 (*P* = 3.961E‐09), rs474978 (*P* = 2.990E‐09), rs690118 (*P* = 2.925E‐09), and rs690253 (*P* = 1.458E‐09), as well as near the *ZNF618* gene on 9q31.3 (rs1435984417, *P* = 2.541E‐08). All identified loci surpassed the genome‐wide significance threshold (*P* < 5 × 10–8). Association results for these significant SNPs, adjusted for the two critical confounding factors, are detailed in Table 4 and Table S4, with a Manhattan plot provided in Fig. S14. Validation using GCTA fastGWA and REGENIE, which controlled for genetic relationships among subjects and case–control imbalances, replicated the significant associations at all identified loci at the genome‐wide significance level, as shown in Fig. S15. The Q statistics and *I* ^2^ values for all SNPs were zero in each TMM‐V2, TMM‐NEO, and NGO‐NEO cohort, indicating no heterogeneity within the cohorts. In addition, the *I* ^2^ values for the SNPs were zero or negligible (<25) in the meta‐analysis of the TMM‐V2, TMM‐NEO, and NGO‐NEO cohorts, indicating no or low heterogeneity among the three cohorts, as shown in Tables S3 and S4.

**Table 4:** SNPs significantly associated with postpartum depression in the meta‐analysis of TMM‐V2, TMM‐NEO, and NGO‐NEO cohorts considering the two most important confounding factors

SNP ID	BP	Chr#	Locus	Gene	EAF	*P*‐value
rs377546683	58,800,275	1	p32.2	*DAB1*	0.02	3.72E‐09
rs11940752	115,442,324	4	q26	*UGT8*	0.96	1.36E‐08
rs141172317	169,216,010	5	q35.1	*DOCK2*	0.02	1.66E‐08
rs117928019	169,226,605	5	q35.1	*DOCK2*	0.02	1.49E‐09
rs76631412	169,259,673	5	q35.1	*DOCK2*	0.02	1.99E‐09
rs118131805	169,267,720	5	q35.1	*DOCK2*	0.02	1.99E‐09
rs188907279	125,991,737	8	q24.13	*ZNF572*	0.02	4.08E‐08
rs504378	93,336,214	9	q22.2	*DIRAS2*	0.10	4.12E‐09
rs690150	93,359,638	9	q22.2	*DIRAS2*	0.10	2.84E‐09
rs491868	93,349,476	9	q22.2	*DIRAS2*	0.10	4.24E‐09
rs689917	93,359,512	9	q22.2	*DIRAS2*	0.10	3.96E‐09
rs474978	93,352,826	9	q22.2	*DIRAS2*	0.10	2.99E‐09
rs690118	93,356,340	9	q22.2	*DIRAS2*	0.11	2.93E‐09
rs690253	93,352,854	9	q22.2	*DIRAS2*	0.10	1.46E‐09
rs1435984417	113,929,504	9	q31.3	*ZNF618*	0.02	2.54E‐08
rs57705782	7,451,115	18	p11.23	*PTPRM*	0.80	3.26E‐09
rs185293917	39,604,656	22	q13.1	*PDGFB*	0.01	1.89E‐09

The single nucleotide polymorphisms (SNPs) listed in the table were significantly associated with postpartum depression in the meta‐analysis of Tohoku Medical Megabank Organization perinatal women sub‐cohort genotyped by the Japonica Array version 2 (TMM‐V2), Tohoku Medical Megabank Organization perinatal women sub‐cohort genotyped by the Japonica Array version NEO (TMM‐NEO), and Nagoya University perinatal women cohort genotyped by the Japonica Array version NEO (NGO‐NEO) considering the two most important confounding factors.

BP, base pair position; Chr #, chromosome number; EAF, effect allele frequency.

### SNP heritability

The results showed that h^2^_SNP_ was reported through the linear mixed model based on an estimate of the risk of PPD in pregnant women. A linear mixed model was used for the analysis of binary traits. The important confounding factors were used as covariates. In the current study, the results indicated that h^2^_SNP_ was 0.36 (SE = 0.02).

### Linkage disequilibrium and functional annotations of SNPs

To check the functionality of the associated SNPs, regional association plots and functional annotations were made for the associated SNPs. The regional association plots for rs188907279, rs57705782, rs377546683, rs138021793, rs11940752, rs141172317, rs117928019, rs76631412, rs118131805, rs185293917, rs504378, rs690150, rs491868, rs689917, rs474978, rs690118, rs690253, and rs1435984417 are shown (Figs S16–S31); some linkage disequilibrium was seen between marker SNPs and others. Fine‐mapping utilizing functional annotations is shown in Table S5.

### Pathway analysis

**Fig. 2:**
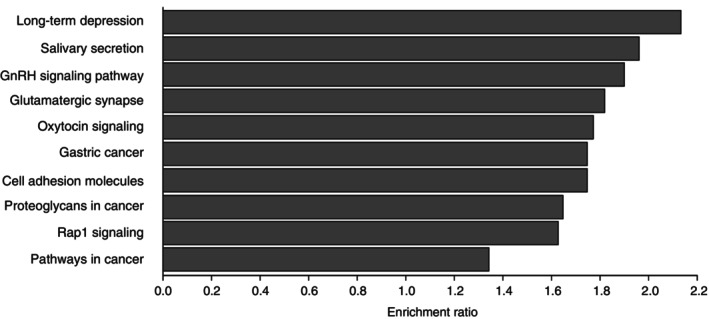
The top 10 pathways in which postpartum depression‐associated genes were overrepresented. The figure shows the top 10 overrepresented pathways with their enrichment ratio based on the WEB‐based Gene Set Analysis Toolkit with Over‐Representation Analysis to identify pathways defined by the KEGG functional database, to which genes belong, where single nucleotide polymorphism suggestively associated with postpartum depression (*P* < 1e‐03) located.

The pathway analysis indicated that the 6049 SNPs suggestively associated with PPD (*P* < 1e‐03) were significantly over‐represented in 46 pathways (*P*‐value < 0.01, FDR <0.05) as listed in Table S6. Of them, pathways with the top 10 enrichment ratio (the observed number of genes suggestively associated with PPD to the expected number of genes based on the KEGG pathway database) included long‐term depression, salivary secretion, gonadotropin‐releasing hormone (GnRH) signaling, glutamatergic synapse, oxytocin signaling, cell adhesion molecules (CAMs), Rap1 signaling, and cancer‐related pathways (Fig. 2).

## Discussion

In this study, GWAS analyses based on TMM‐V2, TMM‐NEO, and NGO‐NEO cohorts, as well as the meta‐analysis, initially failed to detect SNPs with genome‐wide significant associations with PPD when only age and PCA were considered as confounders. This lack of significant findings could stem from the substantial heterogeneity inherent to PPD, notably influenced by differences between primiparous and multiparous women and various psychosocial factors such as marital status, living arrangements, income, educational background, family size, and conception methods. By incorporating seven additional potential confounding factors into the GWAS, SNPs with genome‐wide significant associations were identified at two loci in TMM‐V2 (located at 8q24.13 and 18p11.23) and five loci in TMM‐NEO (located at 1p32.2, 3q25.1, 4q26, 5q35.1, and 22q13.1). Further meta‐analyses across the three cohorts showed significant associations at eight loci (located at 1p32.2, 4q26, 5q35.1, 8q24.13, 9q22.2, 9q31.3, 18p11.23, and 22q13.1), marking the first identification of SNPs associated with PPD at a genome‐wide significant level. These findings underscore the critical influence of psychosocial factors on genetic susceptibility to PPD, showing that such associations are obscured unless comprehensive confounding factors are considered.

While acknowledging the critical role of controlling psychosocial confounders in detecting genetic factors of PPD, the need to exclude subjects with incomplete questionnaire data likely compromised the statistical power of the present GWAS. The present GWAS focused on the most influential confounders to enhance detection power, reducing data exclusions from incomplete responses. Prior GWAS support the significance of this approach of PPD integrating diverse cohorts from European, East Asian, and African ancestries,^35^, ^36^ where larger sample sizes in meta‐analyses did not yield significant associations. Similarly, the present initial analyses, without considering confounders, failed to uncover significant genetic associations, suggesting that factors such as the number of deliveries (i.e., primipara or multipara) and varying family conditions significantly affect genetic susceptibility to PPD. Further refining the GWAS by incorporating the two most critical confounders revealed significant associations at two loci (located at 8q24.13 and 18p11.23) in the TMM‐V2 cohort and five loci (located at 1p32.2, 3q25.1, 4q26, 5q35.1, and 22q13.1) in the TMM‐NEO cohort. A subsequent meta‐analysis across all three cohorts highlighted eight loci with genome‐wide significant associations (located at 1p32.2, 4q26, 5q35.1, 8q24.13, 9q22.2, 9q31.3, 18p11.23, and 22q13.1). These findings underscore the importance of precise confounder control in unveiling the genetic architecture of PPD.

Over the past decade of performing the GWAS to various study designs, some factors, such as binary traits with unbalanced case–control ratios, population stratification, and relatedness,^49^ have been found to inflate GWAS test statistics, and thereby spurious associations such as an inflated false‐positive rate. Therefore, the mixed linear model‐based tool (GCTA fastGWA) and the whole genome regression modeling (REGENIE) were applied to examine our data set. The results indicated that spurious associations due to the above factors did not majorly affect the present findings.

The GWAS analyses of the three cohorts (TMM‐V2, TMM‐NEO, and NGO‐NEO) did not have any genome‐wide significant loci in common. However, the loci with genome‐wide significance in one of the cohorts showed positive associations with PPD in the others, even though they did not reach genome‐wide significance levels, as shown in Tables 1, 2, 3, 4. Although none of the cohorts had sufficient power to detect replicable genetic factors associated with PPD, the meta‐analysis successfully enabled the detection of loci significantly associated with PPD.

Interestingly, the present study revealed that many loci associated with PPD are proximal to genes linked to psychiatric conditions such as MDD, BD, SCZ, attention‐deficit/hyperactivity disorder (ADHD), autism spectrum disorder (ASD), and PTSD,^60^, ^61^, ^62^, ^63^, ^64^ which suggested potential genetic overlaps between PPD and other psychiatric disorders. The details of the links between the associated loci and psychiatric phenotypes were summarized in the supplementary text. Regarding clinical entities, PPD is classified as MDD with onset within 1 month postpartum. Furthermore, PPD shares standard clinical features with BD and SCZ during the postpartum period. Previous research endorsed shared pathogenetic mechanisms between PPD and other psychiatric conditions. Epidemiological data suggested that women with a history of depression were over 20 times more likely to develop PPD.^64^, ^65^, ^66^, ^67^ Research by Chen^60^ and Kiewa *et al*.^68^ has shown that both PPD and postpartum psychosis significantly increase the risks of MDD, BD, and SCZ in mothers and are linked to higher risks of ADHD and ASD in offspring. A meta‐analysis reinforced the association between PPD and ADHD,^69^ whereas conditional process modeling linked emergency cesarean sections to increased PPD risks, potentially mediated by PTSD.^70^ A systematic review of 17,675 women identified prior depression, anxiety, and mental illness as predictors of PTSD post‐childbirth.^71^ Collectively, these findings underscore the complex interrelations and shared genetic susceptibilities between PPD and multiple psychiatric disorders.

Of the psychiatric disorders, MDD and BD may have a higher commonality with PPD; therefore, common genetic variants may be associated with MDD/BD and PPD. While previous GWAS studies^29^, ^30^ of PPD have failed to detect significant associations between genetic loci and PPD, they demonstrated significant genetic correlations between PPD and other psychiatric disorders, including MDD and BD. Of 10 GWAS studies of MDD or depression, two found an association between *DOCK2* and MDD/depression, and four indicated loci in *UGT8*, *PTPRM*, or *DAB1* as variants associated with MDD/depression. Besides these 10 studies, there have been five GWAS studies of BD, of which, one found *DIRAS2* and the other indicated *DOCK2* as variants associated with BD. These findings may support the notion of overlaps in genetic predisposition and pathogenesis between MDD/BD and PPD (Table S7).

Finally, pathway analysis indicated that SNPs suggestively associated with PPD (*P* < 1e‐03) were significantly over‐represented in pathways involved in long‐term depression, salivary secretion, GnRH signaling, glutamatergic synapse, oxytocin signaling, cell adhesion molecules (CAMs), Rap1 signaling, and cancer‐related pathways. Of the pathways, CAMs and Rap1 signaling pathways included three of the eight SNPs, DOCK2, PTPRM, and PDGFB, which showed genome‐wide significant association with PPD. CAMs has been found to be associated with normal pregnancy, preeclampsia, and fetal growth restriction.^72^ Rap1 was reported to be related to depression^73^ and recurrent pregnancy loss.^74^ The other pathways, including long‐term depression in synaptic transmission,^75^ GnRH secretion, salivary secretion,^76^ glutamatergic synapse,^77^, ^78^ and oxytocin signaling^79^ have essential roles in neurotransmission and a variety of reproductive phenotypes, which can be relevant to depressive‐like behaviors in PPD.^80^

This study had several limitations. First, the phenotypic definition of PPD was based only on self‐report questionnaires; clinical diagnoses based on examinations by physicians were not confirmed. Second, although the sample sizes of the two ToMMo cohorts were the largest ever as GWAS studies of PPD, an even larger sample size is required to increase the statistical power needed to detect genetic variants associated with PPD. This study should be replicated with independent equivalent or larger cohorts. Third, caution is needed in interpreting the results because this study was based on cohorts constructed from specific regions of Japan; the findings need to be validated in other regions and ethnicities. In recent years, GWAS data of various phenotypes have been accumulated worldwide and used for risk prediction and gene exploration through meta‐analyses combined with other studies. Although the sample size in this study was not large, the data will be disclosed in the ToMMo database jMorp. They could contribute to the elucidation of PPD as additional studies are accumulated.

In conclusion, the present study highlighted the number of deliveries and the number of family members living together as two critical confounding factors in elucidating the genetic underpinnings of PPD and identified significant associations between PPD and eight loci at DAB1, UGT8, DOCK2, ZNF572, DIRAS2, ZNF618, PTPRM, and PDGFB. These findings illuminate the genetic architecture underlying PPD's pathogenesis.

## Disclosure statement

Norio Ozaki and Hiroaki Tomita are members of the Editorial Board of Psychiatry and Clinical Neurosciences. The authors declare no other competing interests.

## Author contributions

XL took a major role in the data analysis, along with NT, AN, GT, and HT. NT, AN, YN, MsY, KM, MI, TO, MK, FU, HM, HO, IT, TN, NW, TS, FN, SO, JS, NF, KK, MY, NY, NO, GT, ShK, and HT involved data acquisition. XL, NT, AN, YN, MsY, TN, SO, KK, NO, GT, ShK, and HT handled data management. The data were interpreted by XL, NT, AN, YN, NW, TS, ZY, CO, NK, SaK, TM, TH, MS, NO, GT, ShK, and HT. The manuscript drafting was led by XL and HT. NT, AN, NY, NO, GT, ShK, and HT critically revised the draft. XL, NT, AN, NK, SaK, NY, NO, GT, ShK, and HT were pivotal in the study's conception and design. All listed authors made substantial, direct, and intellectual contributions to the work and approved the final manuscript. The corresponding author had full access to all study data and bore final responsibility for the decision to submit for publication.

## Funding information

This work was supported by a grant from the Strategic Research Program for Brain Sciences from the Japan Agency for Medical Research and Development (AMED) (JP20dm0107099, JP20dk0307077, JP21dk0307103), the TMM from the Ministry of Education, Culture, Sports, Science and Technology of Japan, and AMED (JP20km0105001, JP20km0105002).

## Code availability

Descriptions regarding the essential codes used in this study are shown in the supplementary material.

## Supporting information


**Data S1.** Supporting information.

## Data Availability

The datasets generated during and/or analyzed during the current study are available from the corresponding author on reasonable request.
