# Supplementary material for: Identification of risk loci for postpartum depression in a genome‐wide association study
Source: Psychiatry Clin Neurosci. 2024 Sep 17;78(11):712–20. doi: 10.1111/pcn.13731 (PMC11804921; doi:10.1111/pcn.13731)
Supplement: Supplementary file 1 — Data S1. Supporting information. [file PCN-78-712-s001.docx]

**Supplementary Material**

Supplementary Methods 4

Discussion 5

Supplementary Fig.1. Manhattan plot of the association study from TMM-V2 with PCA and age 9

Supplementary Fig.2. Manhattan plot of the association study from TMM-NEO with PCA and age10

Supplementary Fig.3. Manhattan plot of the association study from NGO-NEO with PCA and age11

Supplementary Fig.4. Manhattan plot of the meta-analysis of participants in TMM-V2, TMM-NEO, and NGO-NEO with PCA and age 12

Supplementary Fig.5. Manhattan plot of the association study from TMM-V2 considering factors related to PPD13

Supplementary Fig.6. Manhattan plot of the association study from TMM-NEO considering factors related to PPD14

Supplementary Fig.7. Manhattan plot of the association study from NGO-NEO considering factors related to PPD15

Supplementary Fig.8. Manhattan plot of the meta-analysis of participants in TMM-V2, TMM-NEO, and NGO-NEO considering factors related to PPD 16

Supplementary Fig.9. Manhattan plot of the association study from TMM-V2 considering important factors related to PPD 17

Supplementary Fig.10. Manhattan plot of the association study fromTMM-NEO considering important factors related to PPD18

Supplementary Fig.11. Manhattan plot of the association study from NGO-NEO considering important factor related to PPD 19

Supplementary Fig.12. Manhattan plot of the meta-analysis of participants in TMM-V2, TMM-NEO, and NGO-NEO considering important factors related to PPD 20

Supplementary Fig.13. Manhattan plot of the meta-analysis of participants in TMM-V2, TMM-NEO, and NGO-NEO with PCA and age based on fastGWA and REGENIE21

Supplementary Fig.14. Manhattan plot of the meta-analysis of participants in TMM-V2, TMM-NEO, and NGO-NEO considering factors related to PPD based on fastGWA and REGENIE22

Supplementary Fig.15. Manhattan plot of the meta-analysis of participants in TMM-V2, TMM-NEO, and NGO-NEO considering important factors related to PPD based on fastGWA and REGENIE23

Supplementary Fig.16. Regional association plot for rs37754668324

Supplementary Fig.17. Regional association plot for rs1194075225

Supplementary Fig.18. Regional association plot for rs14117231726

Supplementary Fig.19. Regional association plot for rs11792801927

Supplementary Fig.20. Regional association plot for rs7663141228

Supplementary Fig.21. Regional association plot for rs18890727929

Supplementary Fig.22. Regional association plot for rs50437830

Supplementary Fig.23. Regional association plot for rs69015031

Supplementary Fig.24. Regional association plot for rs49186832

Supplementary Fig.25. Regional association plot for rs68991733

Supplementary Fig.26. Regional association plot for rs47497834

Supplementary Fig.27. Regional association plot for rs69011835

Supplementary Fig.28. Regional association plot for rs69025336

Supplementary Fig.29. Regional association plot for rs143598441737

Supplementary Fig.30. Regional association plot for rs5770578238

Supplementary Fig.31. Regional association plot for rs18529391739

Supplementary Table 1. Associations between PPD phenotype and the TMM (TMM-V2 and TMM-NEO) cohorts-based PPD- associated SNPs in the NGO-NEO cohort considering the multiple potential confounding factors40

Supplementary Table 2. Associations between PPD phenotype and the TMM (TMM-V2 and TMM-NEO) cohorts-based PPD- associated SNPs in the NGO-NEO cohort considering two most important confounding factors41

Supplementary Table 3. SNPs significantly associated with postpartum depression in the meta-analysis of TMM-V2, TMM-NEO, and NGO-NEO cohorts considering the multiple potential confounding factors42

Supplementary Table 4. SNPs significantly associated with postpartum depression in the meta-analysis of TMM-V2, TMM-NEO, and NGO-NEO cohorts considering the two most important confounding factors44

Supplementary Table 5. The results of functional annotations46

Supplementary Table 6. Descriptions and functions of genes48

Supplementary Table 7. PPD in relation to other psychiatric diseases49

References51

**Supplementary Methods**

***Code availability***

Descriptions regarding the essential codes used in this study are shown in the supplementary material.

In the present study, the following links showed the analysis method for each software program:

<https://www.well.ox.ac.uk/~gav/bgen_format/>

<https://www.cog-genomics.org/plink/>

<https://www.cog-genomics.org/plink/2.0/>

<https://genome.sph.umich.edu/wiki/METAL_Documentation>

<https://yanglab.westlake.edu.cn/software/gcta/#Overview>

<https://rgcgithub.github.io/regenie/>

***Detailed procedures of the genome-wide association study***

***Convert the data file types***

Before quality control, the imputed data were converted from ‘. -bgen’ files to hard-called genotypes in PLINK binary format (.bed/.bim/.fam) using PLINK 2.0. ^1^

***Population stratification analysis***

The effects of possible population stratification were corrected using the PLINK 1.9 ^1^ tool and output 10 principal components.

***Genome-wide association analyses***

In this GWAS study, the initial analysis was performed by multivariate generalized logistic regression using PLINK 2.0 ^1^ software with an additive genetic model incorporating seven confounding factors and 10 genetic principal components as covariates. Genetic variants with P < 5e-08 were considered to indicate the genome-wide significance of statistical association, and genetic variants with P < 1e-05 were considered to indicate the suggestive significance of the statistical association. Considering that potential blood relationships among the subjects and unbalanced numbers of case and control groups may have influenced the GWAS results, we used GCTA fastGWA ^2, 3^ and REGENIE ^4^ to evaluate the GWAS of PPD, taking these factors into account.

***Meta-analyses of GWAS and regional association analysis***

Three cohorts (TMM-V2, TMM-NEO, and TMM-NGO) were applied to the logistic regression association analysis using the meta-analysis function in the METAL tool ^5^. The regional association plot was created using LocusZoom. ^6^ Functional annotations of variants of associated loci were performed by SNP nexus. ^7^

***Fine-mapping utilizing functional annotations***

Multi-tissue functional datasets should be useful for fine-mapping causal variants. Regarding the fine-mapping interpretation, we calculated the Combined Annotation-Dependent Depletion (CADD) Phred score, which was used for fine-mapping variant interpretation within specific loci. ^8-10^

***SNP Heritability***

We applied restricted maximum likelihood analysis (REML) in the software tool genome-wide complex trait analysis (GCTA) to estimate the effect of all SNPs which is described as SNP heritability (h^2^_SNP_). We calculated the genetic relationship matrix (GRM) and estimated h^2^_SNP_ in linear mixed model, where measurements of genetic similarity are included as random effects to predict phenotype.

***Pathway analysis***

Genes in which SNPs suggestively associated with PPD (P < 1e-03) were located were subjected to Over-Representation Analysis in the WEB-based Gene Set Analysis Toolkit with Over-Representation Analysis ^11^ to identify pathways defined by KEGG functional database in which genes were involved.

***Prioritization of PPD confounding factors***

***Machine learning algorithms***

Among the confounding factors associated with PPD, the most influential were evaluated by predicting PPD based on those confounding factors using random forest (RF), gradient boosted tree (GBT), and light gradient boosting machine (LGBM) algorithms calculated in Python. In addition, random hyperparameter search cross-validation (RandomizedSearchCV) was alternatively applied to calculate RF, GBT, and LGBM.

***Feature attribution analysis***

To estimate the contributions of confounding factors to PPD, the SHapley Additive exPlanations (SHAP) approach was applied to the feature information. ^12^ For seven potential confounding factors, the mean absolute SHAP value in a specific model, reflecting the mean effect of each feature on the predictions, was calculated. The SHAP value can be considered a feature importance measure.

***Discussion***

Notably, the *DAB1* gene on 1p32.2 has associations with MDD, BD, SCZ, and ASD, *UGT8* on 4q26 with MDD and SCZ, *DOCK2* on 5q35.1 with MDD, SCZ, and ADHD, and *DIRAS2* on 9q22.2 with ADHD. Furthermore, *PTPRM* on 18p11.23 and *PDGFB* on 22q13.1 have been linked to BP and SCZ, respectively. Genetic studies have confirmed these associations, and both altered gene expressions and differential DNA methylations in these loci have been documented in human postmortem brain tissues and blood samples, comparing psychiatric disorders with controls. Behavioral abnormalities in knockout mice models for *DAB1*, *UGT8*, and *DOCK2* also support these findings. These results underline the necessity for further research to confirm the shared genetic bases and establish causal relationships between PPD and these psychiatric disorders.

Of the SNPs linked to PPD, rs377546683, identified at P = 3.85E-08 is situated in the genomic region encoding the *DAB1* gene on 1p32.2. *DAB1* acts as a cytosolic adaptor protein crucial for RELN signaling, a pathway implicated in several psychiatric conditions, including MDD, BD, SCZ, ADHD, and ASD. Tsuneura ^13^ detailed the role of Reelin in neuropsychiatric disorders, noting *DAB1*'s involvement in MDD, BD, and SCZ. In MDD patients, a reduction in Reelin-DAB1 protein expression has been observed in the molecular layer of the hippocampus's dentate gyrus ^14^. Fatemi ^15^ reported lower *DAB1* levels in the hippocampus across MDD, BD, and SCZ cases compared to controls. Studies including a gene-environment interaction analysis have identified *DAB1* as a gene associated with BD, influenced by socioeconomic factors ^16^ , and a family-based study linked RELN genetic variations with increased BD susceptibility in females ^17^. Furthermore, DAB1 has been implicated in SCZ and other psychiatric disorders ^18, 19^. Sánchez-Hidalgo ^20^ reviewed 37 studies, highlighting *DAB1*'s significant association with SCZ, particularly regarding brain morphology and behavioral outcomes. Transient downregulation of *DAB1* during development has demonstrated cognitive impairments and dysfunction in SCZ mouse models ^21^. Imai’s ^22^ behavioral tests on *DAB1* conditional knockout mice underscored the crucial role of Reelin-DAB1 signaling in psychiatric disease pathology, showing symptoms akin to those in humans. In addition, associations between DAB1 and ASD ^23^ have been identified, with reduced DAB1 mRNA levels in the brains of autistic individuals ^24^ and significant links between ASD and *DAB1* SNPs ^25^. A study with Han Chinese participants suggested that the RELN-DAB1 interaction could heighten ASD risk ^26^. Teixeira’s ^27^ experiments with genetically modified mice revealed that *DAB1* downregulation could lead to psychiatric disease-related structural and behavioral deficits. These findings collectively affirm the central role of *DAB1* in the pathogenesis of multiple psychiatric disorders.

UGT8, located near rs11940752 (P = 2.48E-08) on 4q26, has been implicated in MDD and other psychiatric conditions. Aston ^28^ noted reduced UGT8 expression in brain tissues of both MDD patients and controls, suggesting its broader biological role. Hüls ^29^ identified altered methylation at the UGT8 loci in late-life MDD patients, highlighting its epigenetic significance. Le-Niculescu ^30^ found differential UGT8 expression in blood samples from BD patients, validated against animal models and human postmortem brain data, indicating its consistency across different biological matrices. Additionally, Malhotra ^31^ discovered a deletion near the UGT8 gene in schizophrenia (SCZ) patients, correlating with reduced gene expression in early-stage SCZ and implicated in white matter deficits, as evidenced by diminished myelin-associated gene expression including UGT8 ^32, 33, 34^. These findings collectively underscore UGT8’s role across various psychiatric disorders, influencing both gene expression and methylation patterns.

Significant SNPs including rs141172317 (P = 4.61E-08), rs117928019 (P = 6.51E-09), rs76631412 (P = 3.36E-08), and rs118131805 (P = 3.36E-08) are located within the Dedicator of cytokinesis 2 (*DOCK2*) gene on 5q35.1. *DOCK2*, a prominent protein in intracellular signaling, activates Rac G protein isoforms and modulates inflammatory processes in various diseases ^35^. *DOCK2*’s association with psychiatric disorders such as MDD, BD, SCZ, and others has been documented. Joseph ^36^ performed a genome-wide association study of 1,693 MDD cases and 4,506 controls, identifying significant loci, including *DOCK2*. Perez ^37^ reported notable changes in *DOCK2* gene expression in hippocampal tissues of SCZ patients compared to controls. Furthermore, Jensen ^38^ implicated *DOCK2* in ADHD's etiology based on genetic studies. Bainomugisa’s ^39^ research on discordant monozygotic twins suggested a link between *DOCK2* and PTSD, a finding supported by altered methylation in *DOCK2* regions in a genome-wide DNA methylation study of PTSD patients ^40^. In addition, Mehta’s ^41^ analysis of trauma-exposed Vietnam veterans showed altered sperm DNA methylation in *DOCK2* loci, corroborating the gene’s epigenetic variation in psychiatric conditions.

The SNP rs188907279 (P = 4.58E-08) is located in the upstream genome region of the *ZNF572* gene on 8q24.13. To date, no reports have linked *ZNF572* and psychiatric conditions; however, it has been suggested that this gene is involved in maintaining healthy pregnant conditions, because gene expression profiling of second-trimester amniotic fluid identified altered expression of *ZNF572* in the samples from women with spontaneous preterm birth. ^42^

Significant SNPs rs504378 (P = 4.12E-09), rs690150 (P = 2.84E-09), rs491868 (P = 4.24E-09), rs689917 (P = 3.96E-09), rs474978 (P = 2.99E-09), rs690118 (P = 2.93E-09), and rs690253 (P = 1.46E-09) localize near the the *DIRAS2* gene on 9q22.2. A genetic study ^43^ reported associations of *DIRAS2* with BD, ADHD, and other personality disorders ^44^. Further analysis of 600 ADHD patients versus 420 controls linked multiple SNPs and haplotype blocks in *DIRAS2* to ADHD ^43^. Demontis’s ^45^ genome-wide meta-analysis encompassing 20,183 ADHD patients and 35,191 controls highlighted variants at the *DIRAS2* locus, achieving genome-wide significance. Grünewald ^46^ identified 33 genes, including *DIRAS2*, showing significant expression differences in a large ADHD cohort (19,099 cases and 34,194 controls). Grünewald ^47^ also demonstrated that *DIRAS2* expression peaks in the hippocampus and cerebral cortex, with marked increases from prenatal to late postnatal stages in mouse development, underscoring *DIRAS2*'s potential role in ADHD etiology.

rs1435984417 (P = 2.54E-08) is located around the genome region coding the *ZNF618* gene on 9q31.3. However, no obvious link between *ZNF618* on 9q31.3 and psychiatric disorders has been reported.

rs57705782 (P = 3.98E-08) is located near the upstream genome region of the *PTPRM* gene on 18p11.23. The chromosome region 18p11.23 has been reported to be associated with BD and psychosis. ^48-50^ Microarray comparative genomic hybridization of genomic DNA from 150 patients with SCZ and 268 controls indicated an association between the *PTPRM* gene and SCZ. ^51^ In addition, transcriptome studies of postmortem brain tissue and peripheral blood samples indicated that the *PTPRM* transcript was dysregulated in BD. ^30, 52^

rs185293917 (P = 1.52E-08) is located around the genome region coding the *PDGFB* gene on 22q13.1. The chromosome region 22q13 has been reported to be associated with SCZ ^53-55^. A sib-transmission disequilibrium test of 104 small nuclear families segregating SCZ and schizoaffective disorder indicated an association between the *PDGFB* gene and SCZ. ^56^ In addition, a mutation in the *PDGFB* gene was detected in a patient with SCZ with brain calcification. ^57^ DNA methylation profiling of the prefrontal cortex from 335 controls and 191 patients with SCZ indicated altered methylation patterns in the *PDGFB* gene region of SCZ samples. ^58^ The *PDGFB* and *PDGFB* receptors may be important in the pathology of SCZ through interactions with the DRD2/DRD4 and NMDA receptors ^58^.

**Supplementary Fig. 1. Manhattan plot of the association study from TMM-V2 with PCA and age**

**
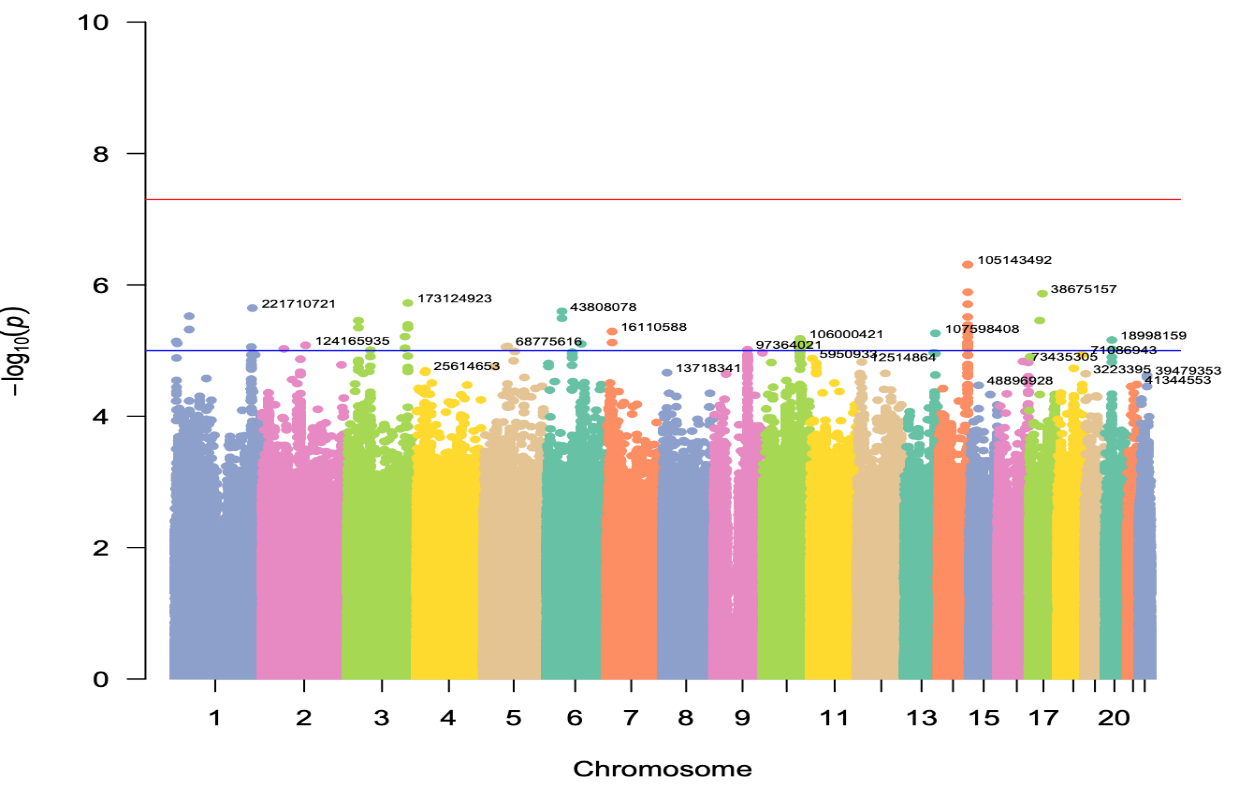
**

**Supplementary Fig. 2. Manhattan plot of the association study from TMM-NEO with PCA and age**

**
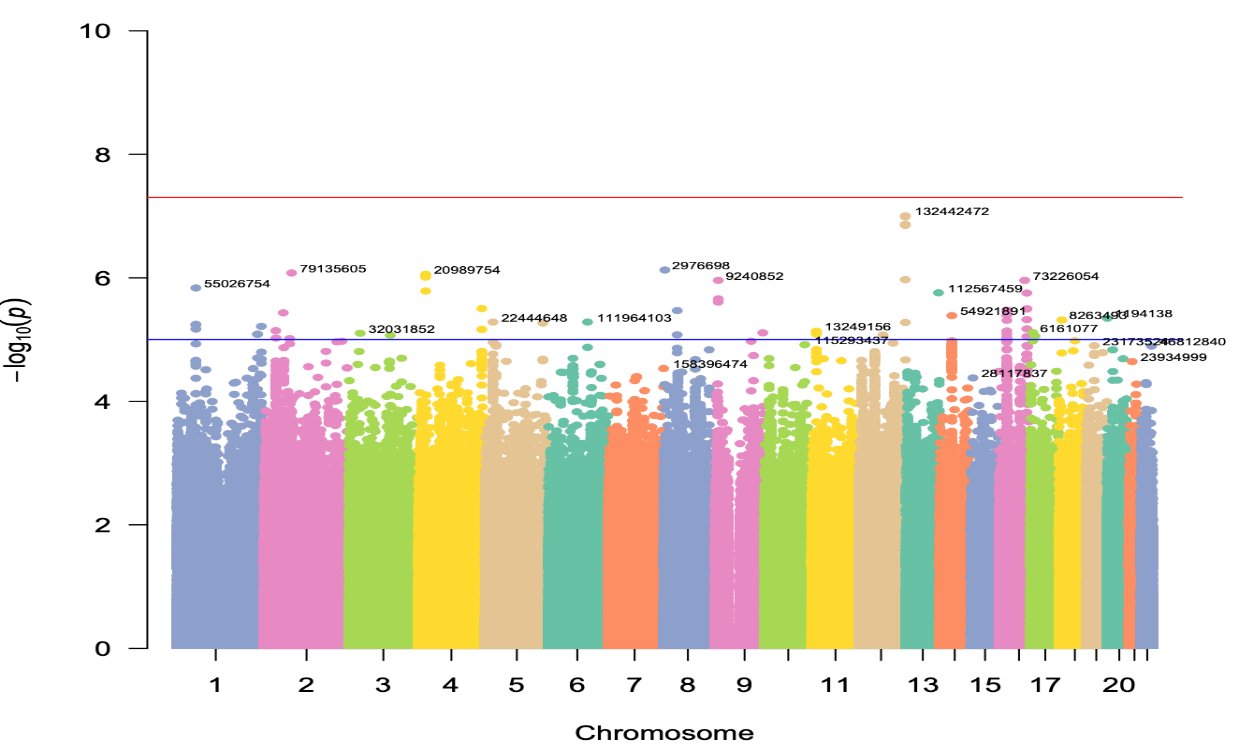
**

**Supplementary Fig. 3. Manhattan plot of the association study from NGO-NEO with PCA and age**

**
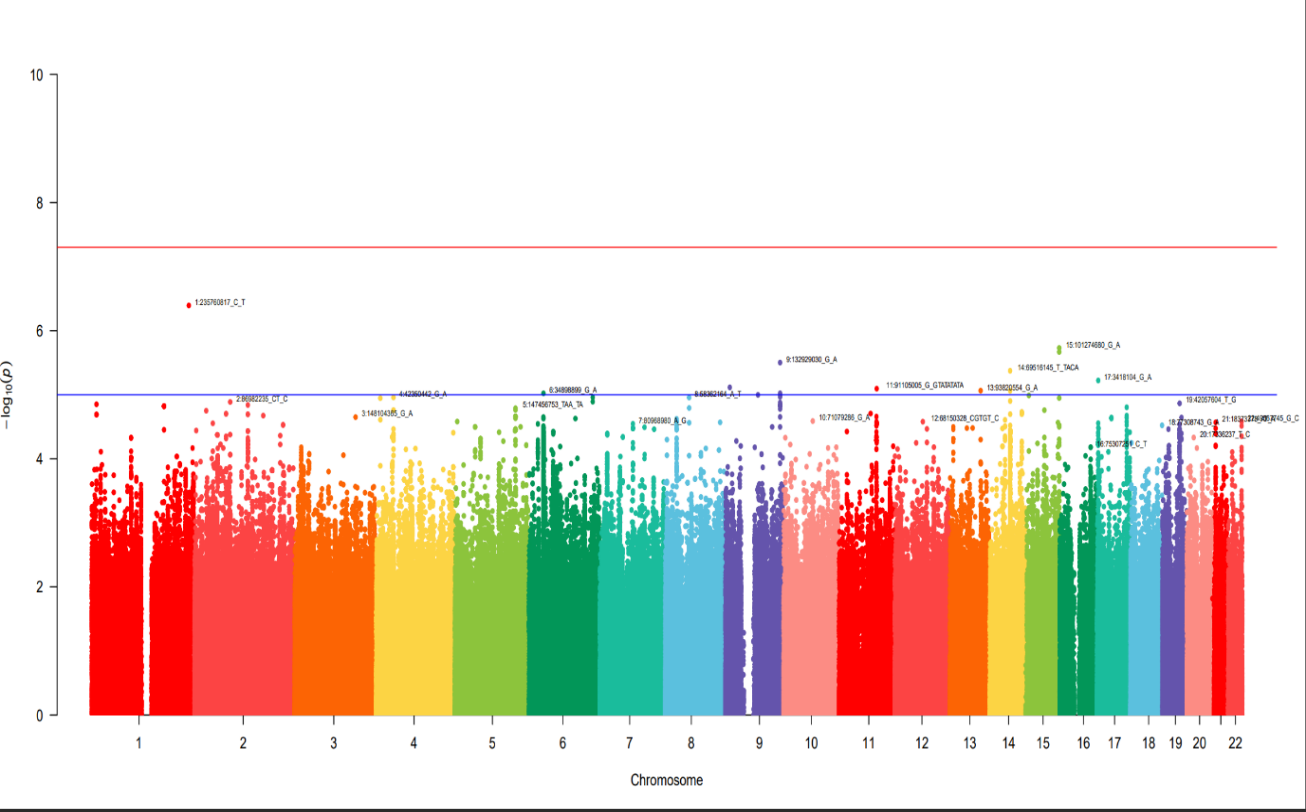
**

**Supplementary Fig. 4. Manhattan plot of the meta-analysis of participants in TMM-V2, TMM-NEO, and NGO-NEO with PCA and age**

**
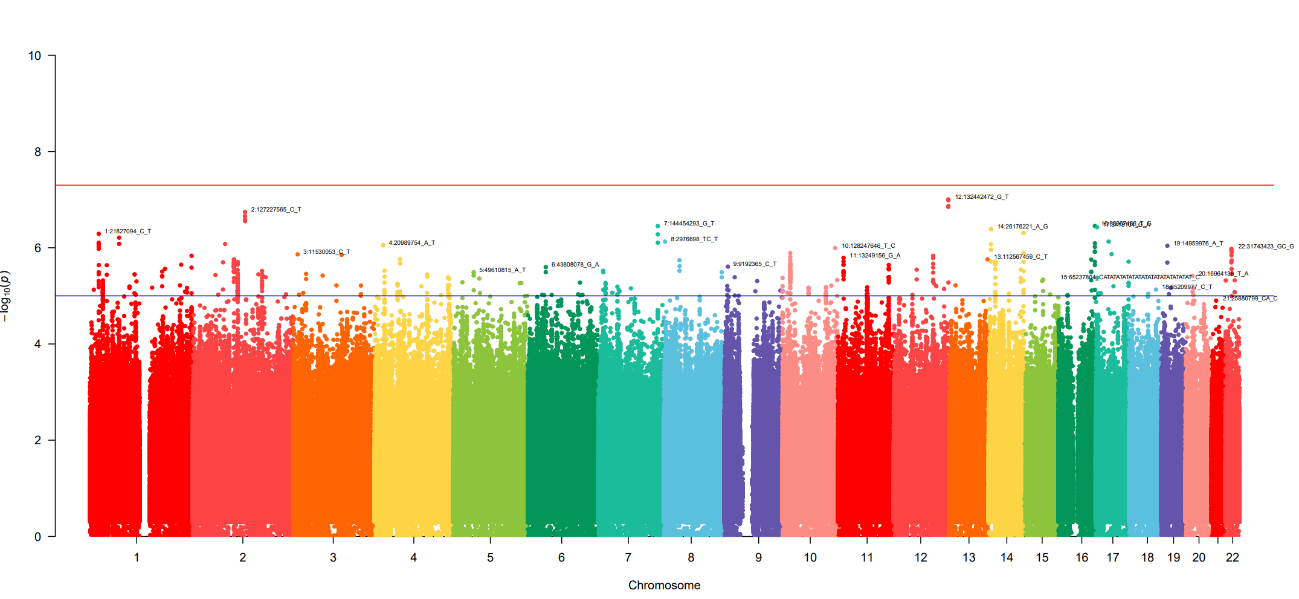
**

**Supplementary Fig. 5. Manhattan plot of the meta-analysis of participants in TMM-V2, TMM-NEO, and NGO-NEO with PCA and age based on fastGWA and REGENIE**

**
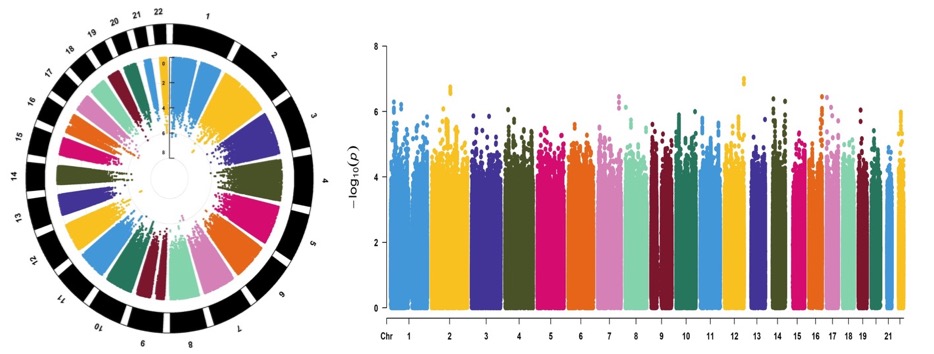
**

**Supplementary Fig. 6. Manhattan plot of the association study from TMM-V2 considering factors related to PPD**

**
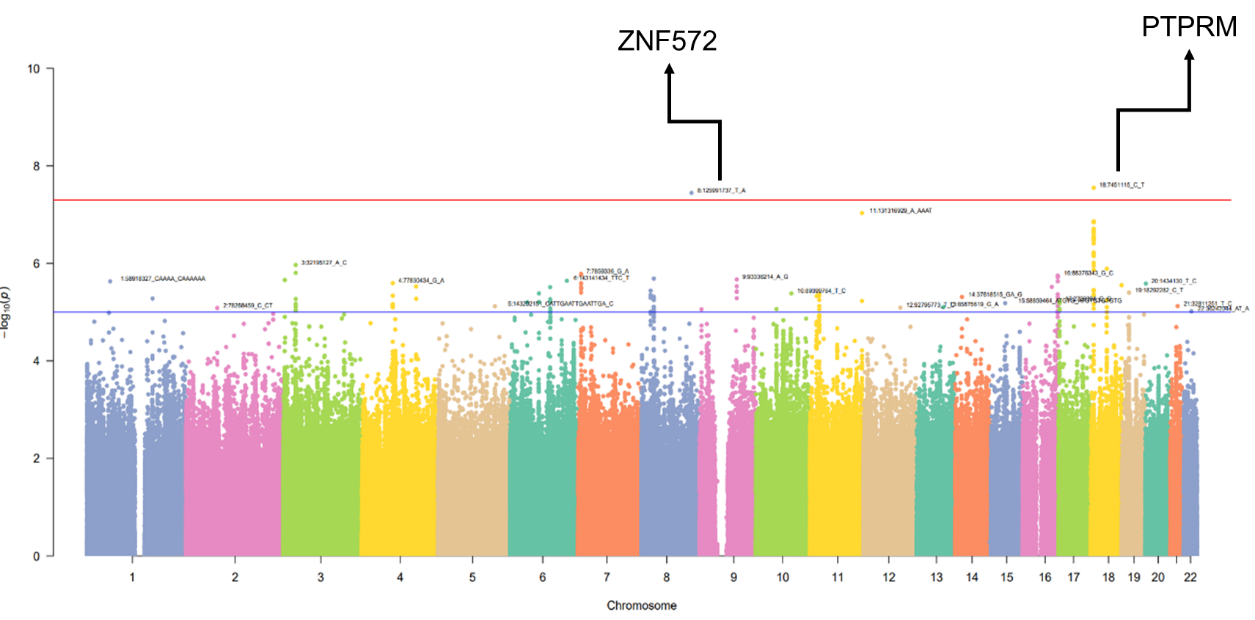
**

**Supplementary Fig. 7. Manhattan plot of the association study from TMM-NEO considering factors related to PPD**

**
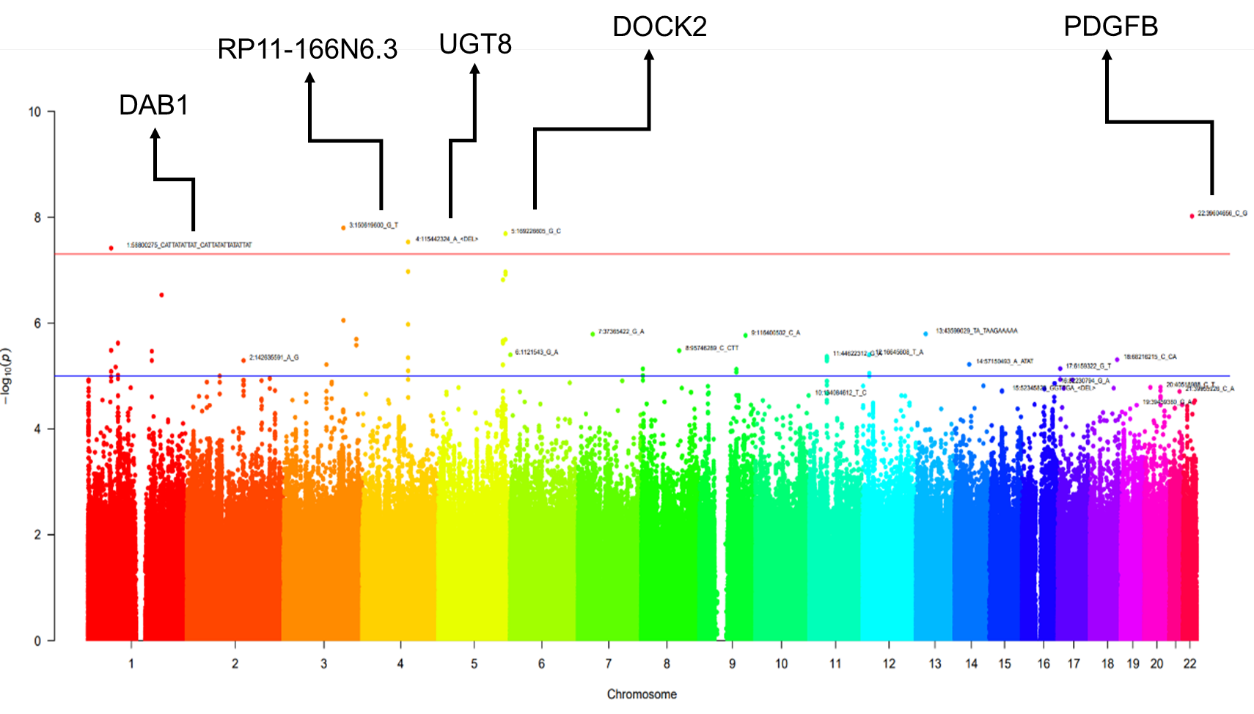
**

**Supplementary Fig. 8. Manhattan plot of the association study from NGO-NEO considering factors related to PPD**

**
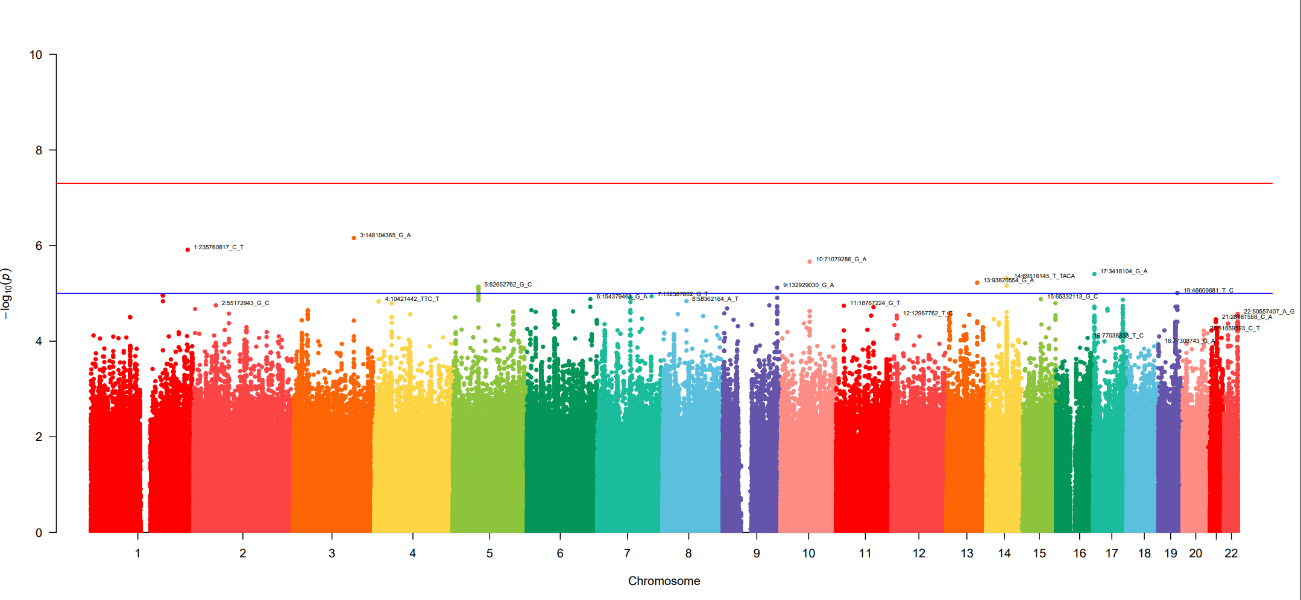
**

**Supplementary Fig. 9. Manhattan plot of the meta-analysis of participants in TMM-V2, TMM-NEO, and NGO-NEO considering factors related to PPD**

**
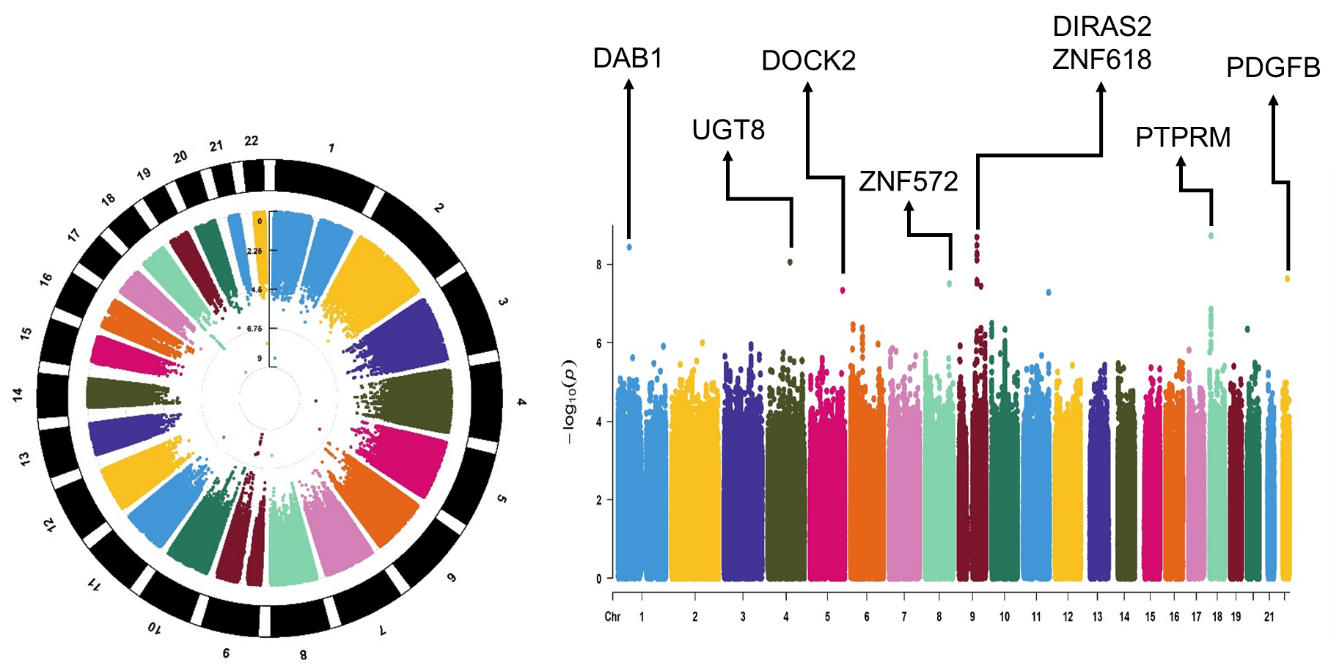
**

**Supplementary Fig. 10. Manhattan plot of the meta-analysis of participants in TMM-V2, TMM-NEO, and NGO-NEO considering factors related to PPD based on fastGWA and REGENIE**

**Supplementary Fig. 11. Manhattan plot of the association study from TMM-V2 considering important factors related to PPD**

**
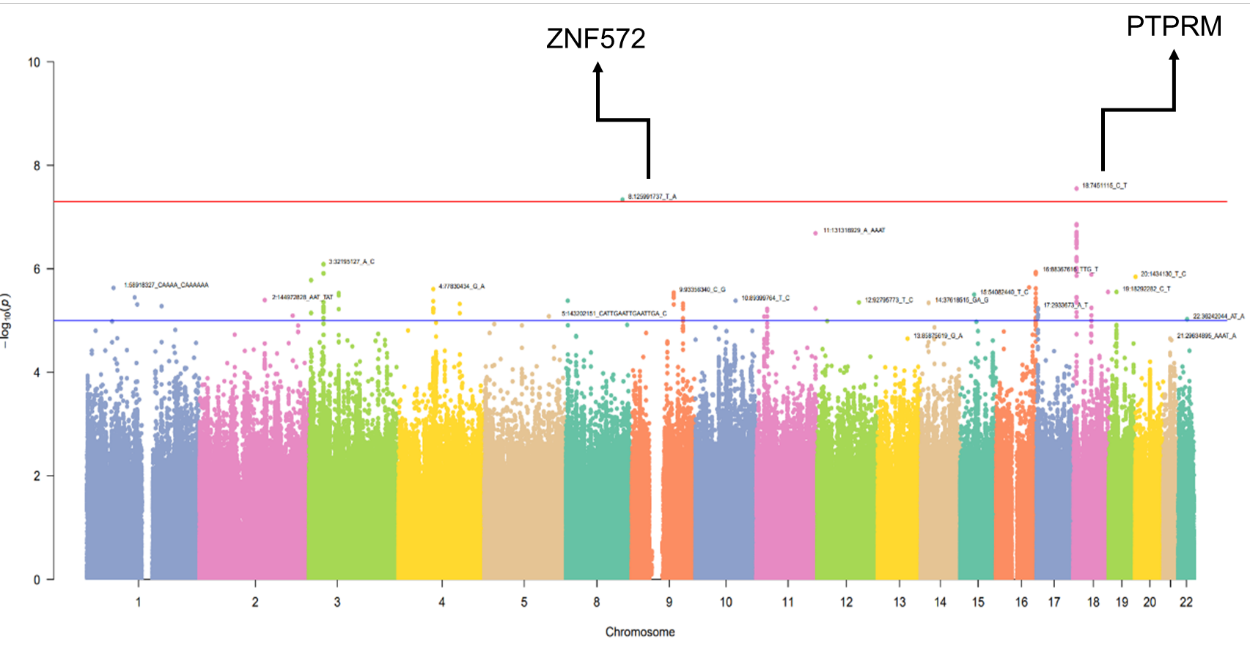
**

**Supplementary Fig. 12. Manhattan plot of the association study from TMM-NEO considering important factors related to PPD**

**
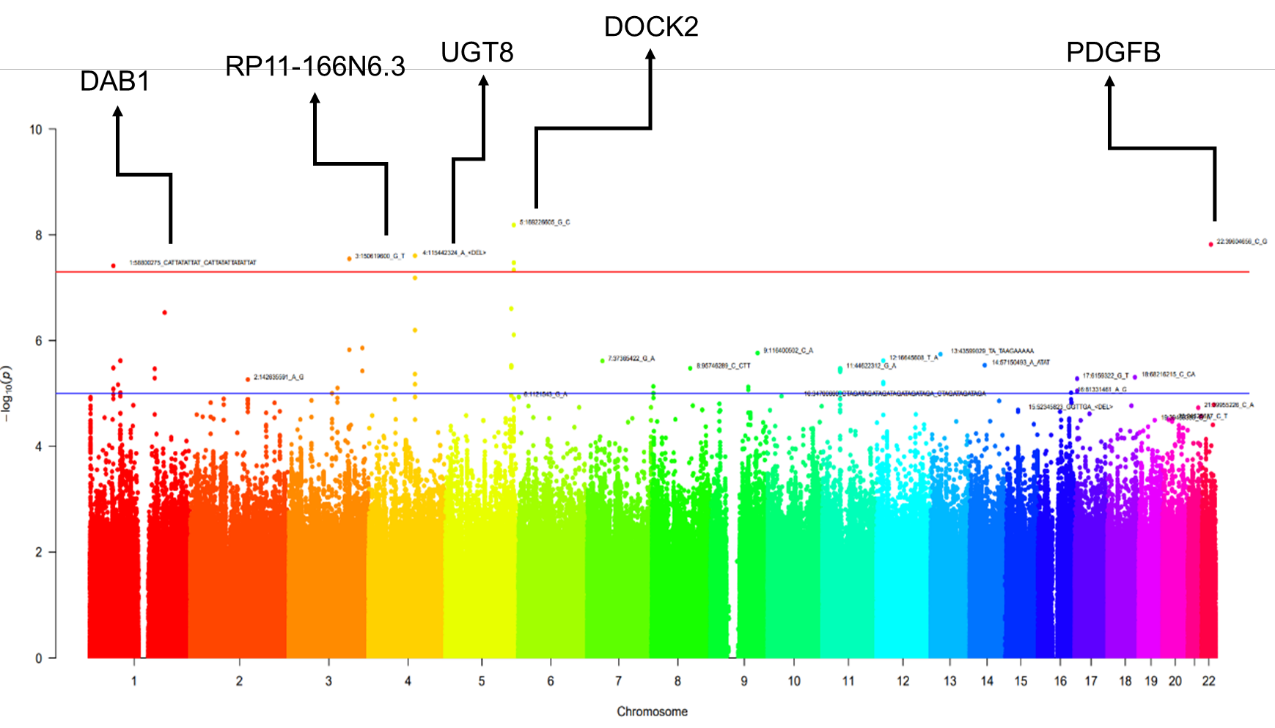
**

**Supplementary Fig. 13. Manhattan plot of the association study from NGO-NEO considering important factor related to PPD**

**
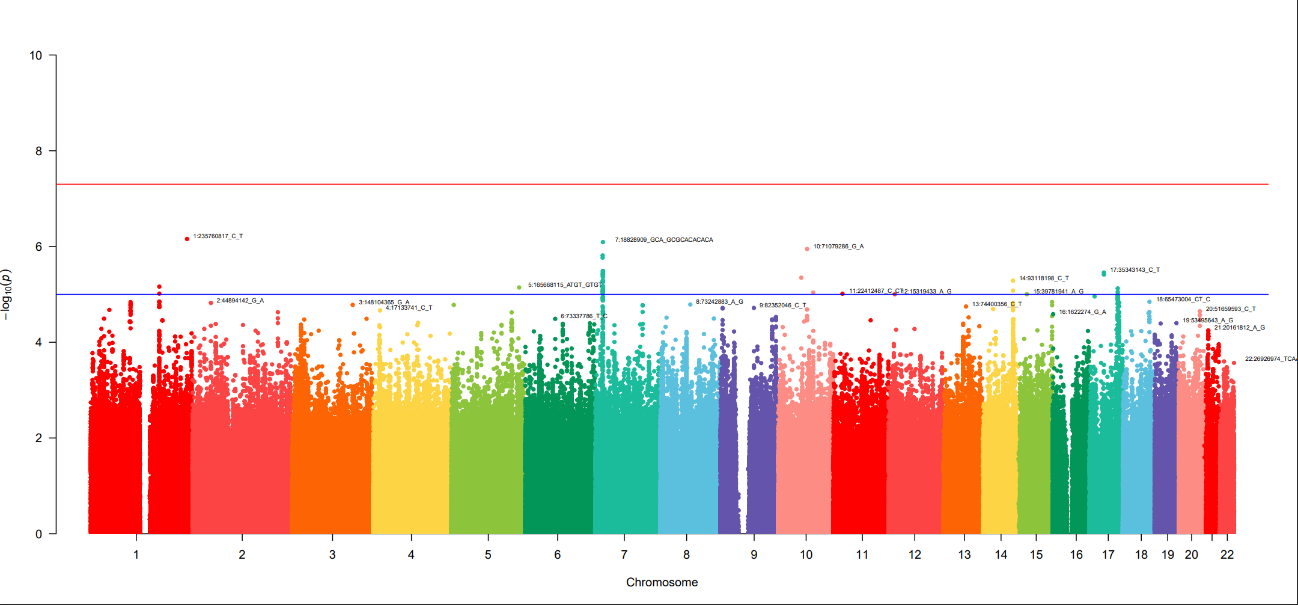
**

**Supplementary Fig. 14. Manhattan plot of the meta-analysis of participants in TMM-V2, TMM-NEO, and NGO-NEO considering important factors related to PPD**


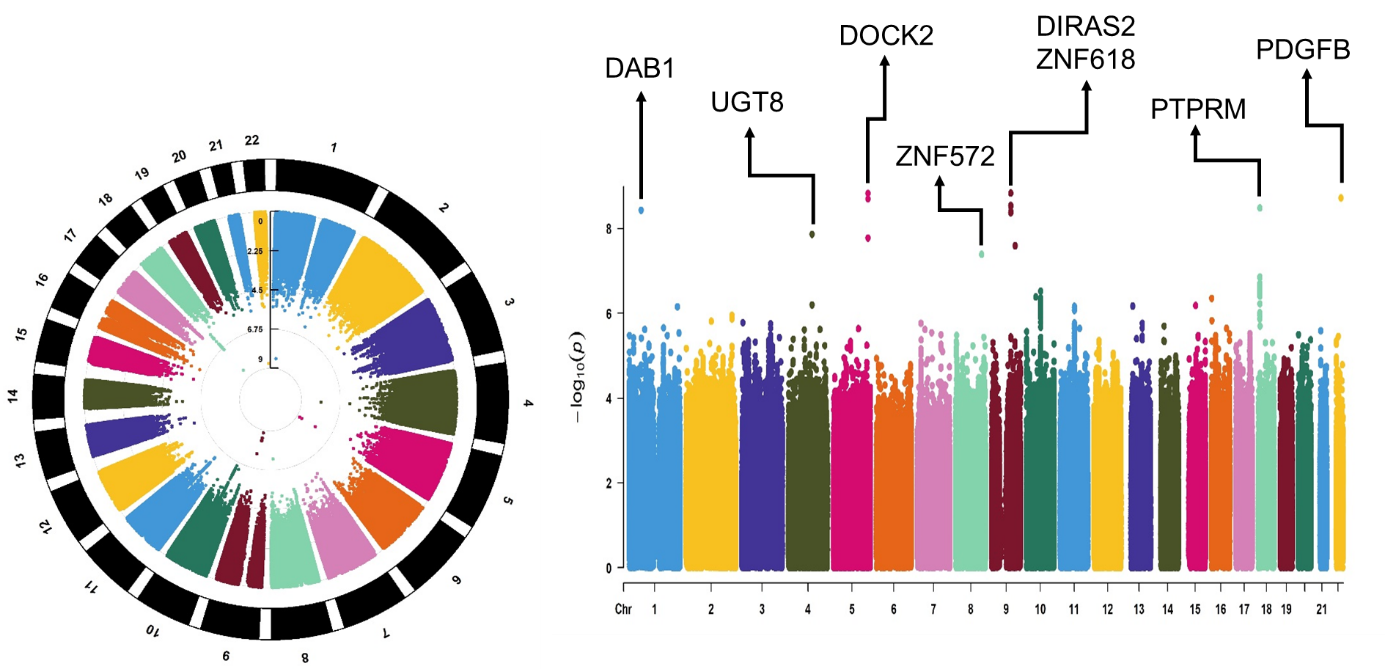


**Supplementary Fig. 15. Manhattan plot of the meta-analysis of participants in TMM-V2, TMM-NEO, and NGO-NEO considering important factors related to PPD based on fastGWA and REGENIE**

**Supplementary Fig. 16. Regional association plot for rs377546683**


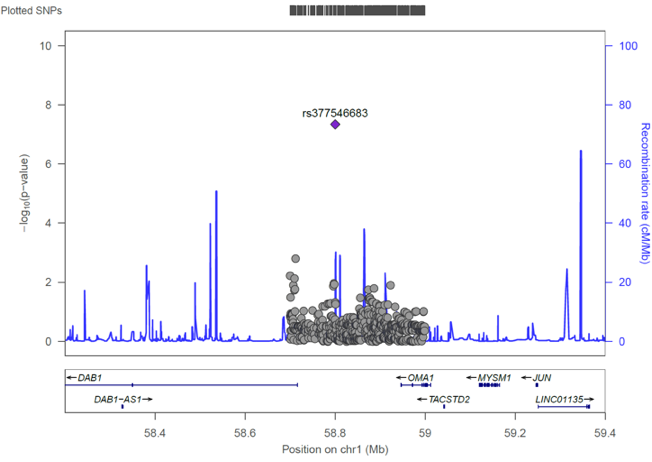


**Supplementary Fig. 17. Regional association plot for rs11940752**


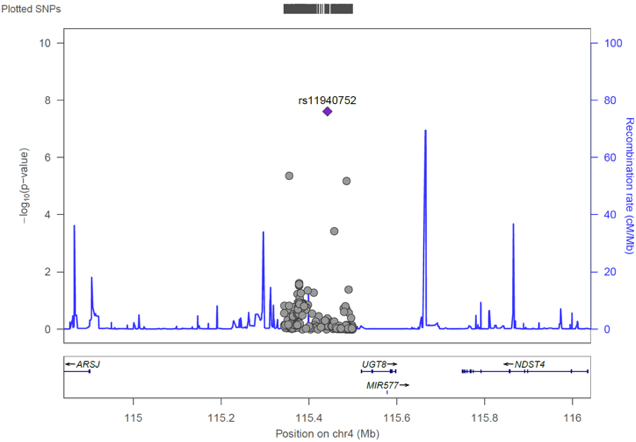


**Supplementary Fig. 18. Regional association plot for rs141172317**


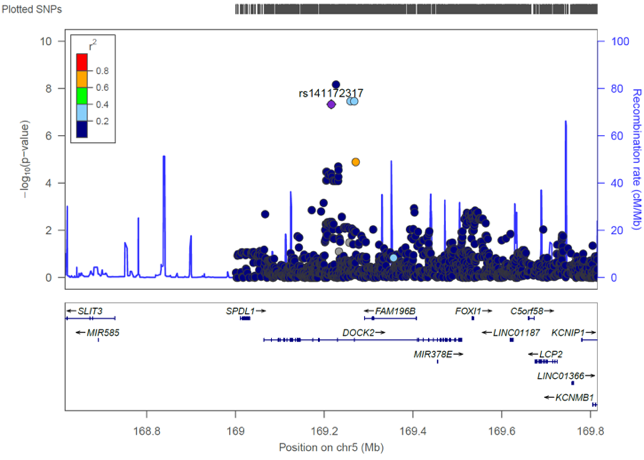


**Supplementary Fig. 19. Regional association plot for rs117928019**


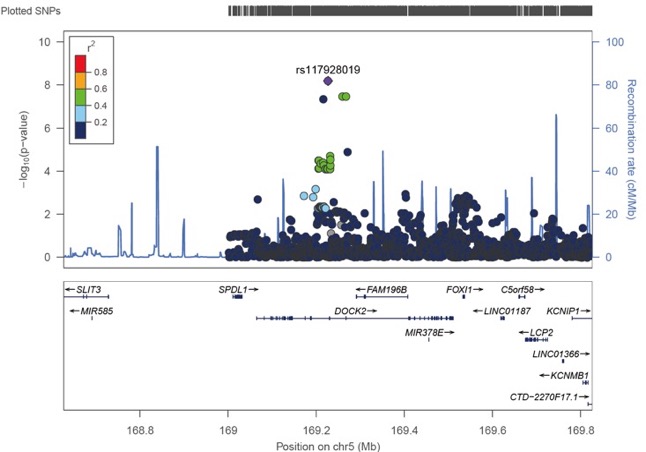


**Supplementary Fig. 20. Regional association plot for rs76631412**


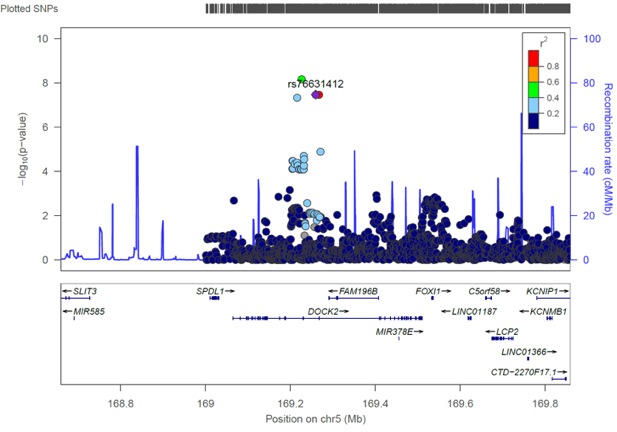


**Supplementary Fig. 21. Regional association plot for rs188907279**


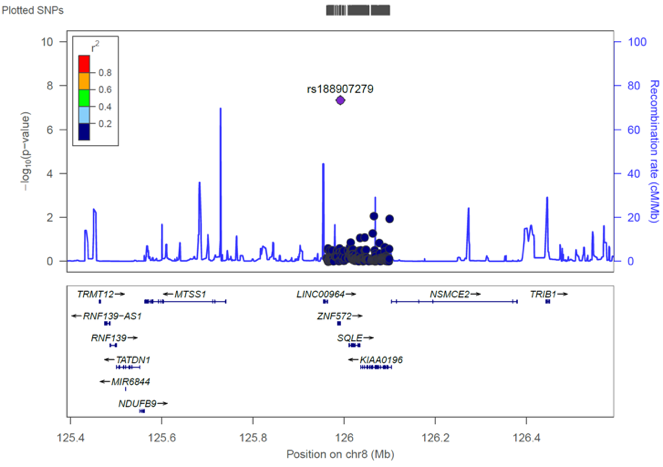


**Supplementary Fig. 22. Regional association plot for rs504378**


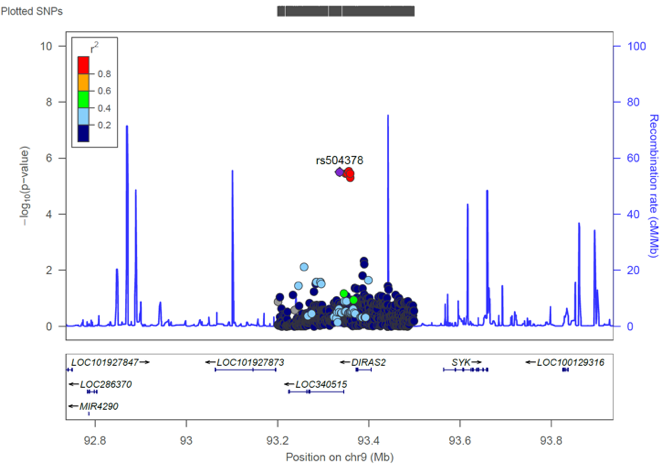


**Supplementary Fig. 23. Regional association plot for rs690150**


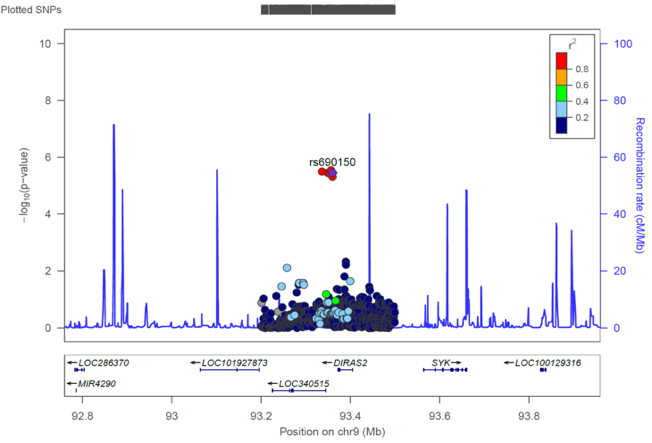


**Supplementary Fig. 24. Regional association plot for rs491868**


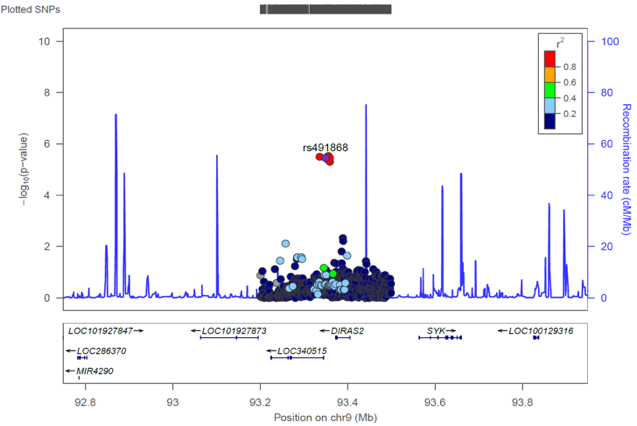


**Supplementary Fig. 25. Regional association plot for rs689917**


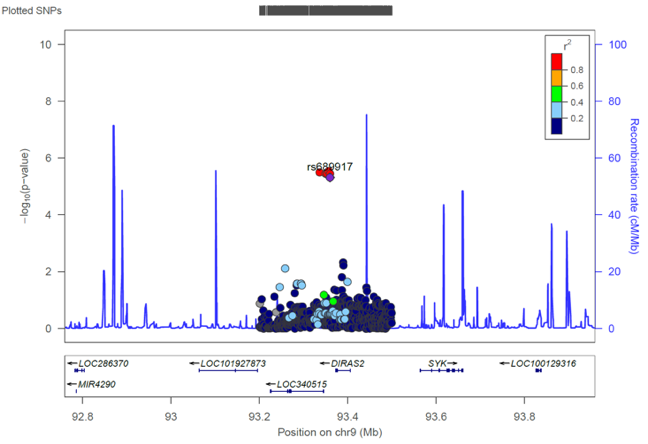


**Supplementary Fig. 26. Regional association plot for rs474978**


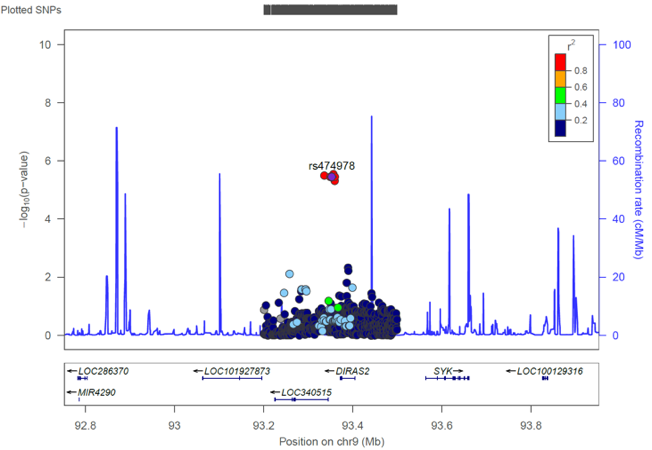


**Supplementary Fig. 27 Regional association plot for rs690118**


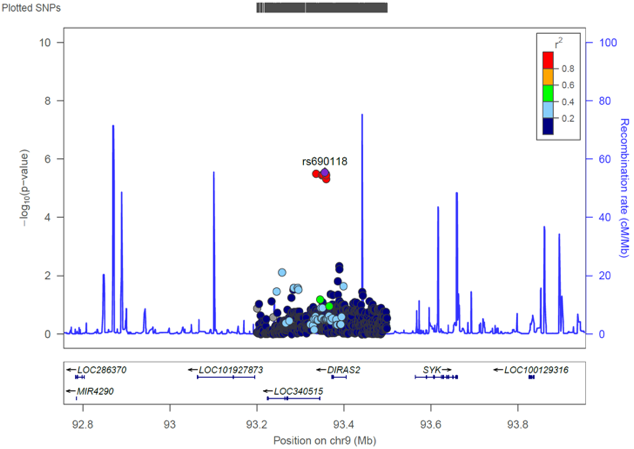


**Supplementary Fig. 28. Regional association plot for rs690253**


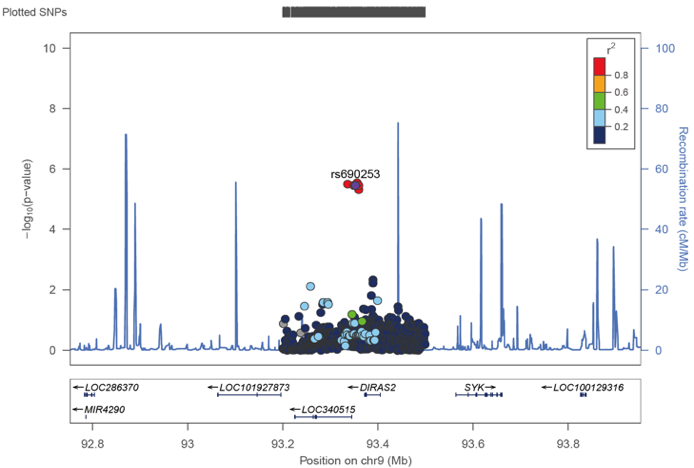


**Supplementary Fig. 29. Regional association plot for rs1435984417**


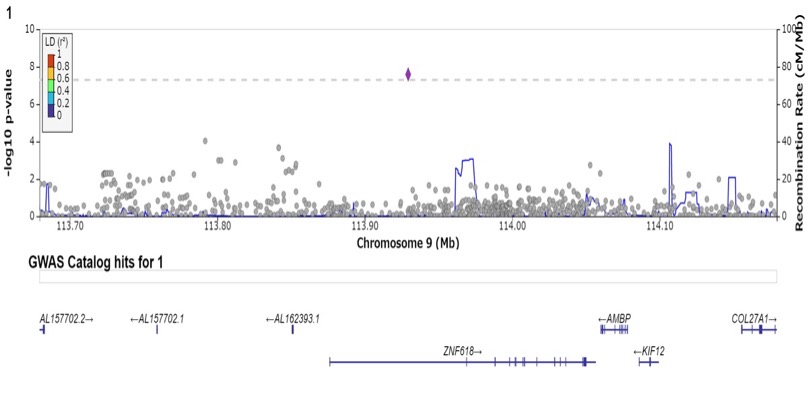


**Supplementary Fig. 30. Regional association plot for rs57705782**


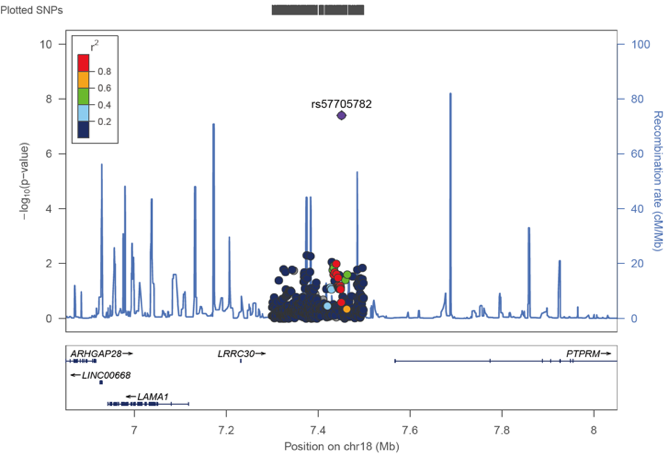


**Supplementary Fig. 31. Regional association plot for rs185293917**

**
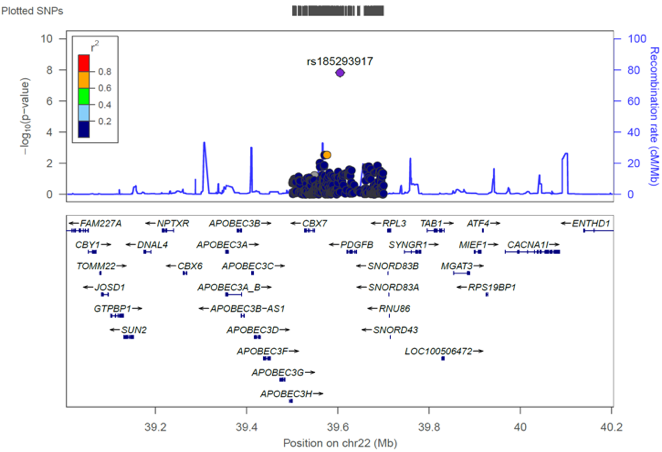
**

**Supplementary Table 1. Associations between PPD phenotype and the TMM (TMM-V2 and TMM-NEO) cohorts-based PPD- associated SNPs in the NGO-NEO cohort considering the multiple potential confounding factors**

| Cohort | SNP ID | BP | Chr# | Locus | Gene | EA/RA | EAF | P value | OR (95% CI) | LOG(OR)_SE | SE |
| --- | --- | --- | --- | --- | --- | --- | --- | --- | --- | --- | --- |
| **NGO-NEO** | rs377546683 | 58800275 | 1 | p32.2 | *DAB1* | C/G | 0.05 | 4.41E-03 | 0.57 (0.05-7.27) | 0.73 | 1.30 |
|  | rs138021793 | 150619600 | 3 | q25.1 | *RP11-166N6.3* | T/G | 0.02 | 0.50 | 0.59 (0.03-11.10) | 0.79 | 1.50 |
|  | rs11940752 | 115442324 | 4 | q26 | *UGT8* | A/T | 0.96 | 2.87E-04 | 2.65 (1.07-6.55) | 0.45 | 0.46 |
|  | rs117928019 | 169226605 | 5 | q35.1 | *DOCK2* | C/G | 0.03 | 8.56E-03 | 0.91 (0.03-5.06) | 0.53 | 5.62 |
|  | rs188907279 | 125991737 | 8 | q24.13 | *ZNF572* | A/T | 0.02 | 6.09E-03 | 1.33 (0.03-5.28) | 0.55 | 1.93 |
|  | rs57705782 | 7451115 | 18 | p11.23 | *PTPRM* | T/C | 0.84 | 6.21E-04 | 0.89 (0.02-5.04) | 0.24 | 2.06 |
|  | rs185293917 | 39604656 | 22 | q13.1 | *PDGFB* | G/C | 0.03 | 6.91E-04 | 1.28 (0.01-6.58) | 0.62 | 2.51 |

The single nucleotide polymorphisms (SNPs) listed in the table were significantly associated with postpartum depression in the Tohoku Medical Megabank Organization perinatal women sub-cohort genotyped by the Japonica Array version 2 (TMM-V2) or the Tohoku Medical Megabank Organization perinatal women sub-cohort genotyped by the Japonica Array version NEO (TMM-NEO) considering the multiple potential confounding factors. The table summarizes information regarding how each SNP is associated with the PPD phenotype in the Nagoya University perinatal women cohort genotyped by the Japonica Array version NEO (NGO-NEO). The Q statistics and I² values for all SNPs were zero in NGO-NEO cohort, indicating no heterogeneity within the cohort.

BP: base pair position, Chr #: chromosome number, EA: effect allele, RA: reference allele, EAF: effect allele frequency, OR (95% CI): odds ratio (95% confidence interval), LOG(OR)_SE: standard error of the log odds ratio, SE: standard error

**Supplementary Table 2. Associations between PPD phenotype and the TMM (TMM-V2 and TMM-NEO) cohorts-based PPD- associated SNPs in the NGO-NEO cohort considering two most important confounding factors**

| Cohort | SNP ID | BP | Chr# | Locus | Gene | EA/RA | EAF | P value | OR (95% CI) | LOG(OR)_SE | SE |
| --- | --- | --- | --- | --- | --- | --- | --- | --- | --- | --- | --- |
| **NGO-NEO** | rs377546683 | 58800275 | 1 | p32.2 | *DAB1* | C/G | 0.05 | 4.50E-03 | 0.64 (0.05-8.92) | 0.60 | 1.34 |
|  | rs138021793 | 150619600 | 3 | q25.1 | *RP11-166N6.3* | T/G | 0.02 | 0.45 | 0.61 (0.05-8.03) | 0.65 | 1.32 |
|  | rs11940752 | 115442324 | 4 | q26 | *UGT8* | A/T | 0.96 | 4.35E-04 | 1.34 (0.11-15.97) | 0.37 | 1.26 |
|  | rs141172317 | 169216010 | 5 | q35.1 | *DOCK2* | T/C | 0.03 | 4.62E-04 | 0.69 (0.05-10.20) | 0.51 | 1.37 |
|  | rs117928019 | 169226605 | 5 | q35.1 | *DOCK2* | C/G | 0.03 | 6.50E-04 | 0.81 (0.01-7.38) | 0.48 | 2.28 |
|  | rs76631412 | 169259673 | 5 | q35.1 | *DOCK2* | A/T | 0.03 | 3.19E-04 | 0.61 (0.08-4.43) | 0.50 | 1.01 |
|  | rs118131805 | 169267720 | 5 | q35.1 | *DOCK2* | T/G | 0.03 | 3.19E-04 | 0.61 (0.08-4.43) | 0.50 | 1.01 |
|  | rs188907279 | 125991737 | 8 | q24.13 | *ZNF572* | A/T | 0.02 | 6.01E-04 | 1.15 (0.01-6.43) | 0.54 | 3.86 |
|  | rs57705782 | 7451115 | 18 | p11.23 | *PTPRM* | T/C | 0.80 | 4.86E-03 | 0.21 (0.07-0.63) | 0.87 | 0.56 |
|  | rs185293917 | 39604656 | 22 | q13.1 | *PDGFB* | G/C | 0.03 | 4.57E-03 | 1.51 (0.11-9.65) | 0.55 | 1.33 |

The single nucleotide polymorphisms (SNPs) listed in the table were significantly associated with postpartum depression in the Tohoku Medical Megabank Organization perinatal women sub-cohort genotyped by the Japonica Array version 2 (TMM-V2) or the Tohoku Medical Megabank Organization perinatal women sub-cohort genotyped by the Japonica Array version NEO (TMM-NEO), considering two most important confounding factors. The table summarizes information regarding how each SNP is associated with the PPD phenotype in the Nagoya University perinatal women cohort genotyped by the Japonica Array version NEO (NGO-NEO). The Q statistics and I² values for all SNPs were zero in NGO-NEO cohort, indicating no heterogeneity within the cohort.

BP: base pair position, Chr #: chromosome number, EA: effect allele, RA: reference allele, EAF: effect allele frequency, OR (95% CI): odds ratio (95% confidence interval), LOG(OR)_SE: standard error of the log odds ratio, SE: standard error

**Supplementary Table 3. SNPs significantly associated with postpartum depression in the meta-analysis of TMM-V2, TMM-NEO, and NGO-NEO cohorts considering the multiple potential confounding factors**

| **Associated SNPs** | | | | | **Meta-analysis** | | **TMM-V2** | | | | **TMM-NEO** | | | | **NGO-NEO** | | | |  | |
| --- | --- | --- | --- | --- | --- | --- | --- | --- | --- | --- | --- | --- | --- | --- | --- | --- | --- | --- | --- | --- |
| SNP ID | BP | Chr# | Locus | Gene | EA/RA | P | P | OR | LOG(OR)_SE | SE | P | OR | LOG(OR)_SE | SE | P | OR | LOG(OR)_SE | SE | Q statistic | I^2^ |
| rs377546683 | 58800275 | 1 | p32.2 | *DAB1* | C/G | 3.62E-09 | 0.08 | 1.07 | 0.06 | 0.81 | 1.60E-08 | 2.78 | 0.18 | 0.18 | 4.41E-03 | 0.57 | 0.73 | 1.30 | 2.70 | 5.97 |
| rs11940752 | 115442324 | 4 | q26 | *UGT8* | A/T | 8.73E-09 | 0.43 | 0.88 | 0.17 | 1.26 | 2.95E-08 | 2.13 | 0.14 | 0.18 | 2.87E-04 | 2.65 | 0.45 | 0.46 | 0.71 | 0 |
| rs117928019 | 169226605 | 5 | q35.1 | *DOCK2* | C/G | 4.61E-08 | 0.22 | 0.77 | 0.21 | 0.81 | 2.05E-08 | 2.62 | 0.17 | 0.18 | 8.56E-03 | 0.91 | 0.53 | 5.62 | 2.21 | 9.48 |
| rs188907279 | 125991737 | 8 | q24.13 | *ZNF572* | A/T | 3.13E-08 | 3.58E-08 | 1.54 | 0.17 | 0.39 | 0.54 | 1.17 | 0.21 | 1.32 | 6.09E-03 | 1.33 | 0.55 | 1.93 | 0.04 | 0 |
| rs504378 | 93336214 | 9 | q22.2 | *DIRAS2* | A/G | 2.02E-09 | 2.13E-06 | 1.19 | 0.09 | 0.49 | 1.85E-04 | 1.19 | 0.09 | 0.51 | 0.08 | 1.60 | 0.26 | 0.56 | 0.20 | 0 |
| rs690150 | 93359638 | 9 | q22.2 | *DIRAS2* | A/G | 5.16E-09 | 3.80E-06 | 1.20 | 0.08 | 0.47 | 1.62E-04 | 1.17 | 0.09 | 0.60 | 0.08 | 1.58 | 0.26 | 0.58 | 0.17 | 0 |
| rs491868 | 93349476 | 9 | q22.2 | *DIRAS2* | T/C | 5.83E-09 | 3.80E-06 | 1.20 | 0.08 | 0.46 | 2.30E-04 | 1.16 | 0.09 | 0.61 | 0.08 | 1.60 | 0.26 | 0.56 | 0.20 | 0 |
| rs689917 | 93359512 | 9 | q22.2 | *DIRAS2* | A/G | 7.84E-09 | 5.18E-06 | 1.19 | 0.08 | 0.48 | 1.84E-04 | 1.16 | 0.09 | 0.60 | 0.09 | 1.57 | 0.26 | 0.59 | 0.17 | 0 |
| rs474978 | 93352826 | 9 | q22.2 | *DIRAS2* | A/T | 5.77E-09 | 3.80E-06 | 1.20 | 0.08 | 0.47 | 1.84E-04 | 1.17 | 0.09 | 0.60 | 0.08 | 1.58 | 0.26 | 0.58 | 0.17 | 0 |
| rs690118 | 93356340 | 9 | q22.2 | *DIRAS2* | C/G | 2.60E-08 | 2.94E-06 | 1.19 | 0.08 | 0.48 | 6.92E-04 | 1.16 | 0.09 | 0.60 | 0.12 | 1.51 | 0.26 | 0.64 | 0.11 | 0 |
| rs690253 | 93352854 | 9 | q22.2 | *DIRAS2* | A/C | 3.25E-09 | 3.80E-06 | 1.19 | 0.08 | 0.48 | 2.21E-04 | 1.17 | 0.09 | 0.59 | 0.05 | 1.66 | 0.26 | 0.52 | 0.28 | 0 |
| rs1435984417 | 113929504 | 9 | q31.3 | *ZNF618* | G/C | 3.56E-08 | 0.003 | 1.34 | 0.18 | 0.63 | 1.90E-04 | 1.63 | 0.20 | 0.41 | 3.96E-03 | 6.03 | 0.62 | 0.35 | 7.83 | 9.45 |
| rs57705782 | 7451115 | 18 | p11.23 | *PTPRM* | T/C | 1.86E-09 | 2.81E-08 | 1.16 | 0.07 | 0.48 | 0.71 | 0.82 | 0.08 | 0.42 | 6.21E-04 | 0.89 | 0.24 | 0.89 | 0.30 | 0 |
| rs185293917 | 39604656 | 22 | q13.1 | *PDGFB* | G/C | 2.33E-08 | 0.59 | 1.14 | 0.24 | 1.84 | 9.58E-09 | 3.25 | 0.21 | 0.17 | 6.91E-04 | 1.28 | 0.62 | 1.28 | 0.83 | 0 |

The single nucleotide polymorphisms (SNPs) listed in the table were significantly associated with postpartum depression in the meta-analysis of Tohoku Medical Megabank Organization perinatal women sub-cohort genotyped by the Japonica Array version 2 (TMM-V2), Tohoku Medical Megabank Organization perinatal women sub-cohort genotyped by the Japonica Array version NEO (TMM-NEO), and Nagoya University perinatal women cohort genotyped by the Japonica Array version NEO (NGO-NEO) considering the multiple potential confounding factors. The I² values for the SNPs were zero or negligible (<25) in the meta-analysis of the TMM-V2, TMM-NEO, and NGO-NEO cohorts, indicating no or low heterogeneity among the three cohorts.

BP: base pair position, Chr #: chromosome number, EAF: effect allele frequency, OR: odds ratio, LOG(OR)_SE: standard error of the log odds ratio, SE: standard error

**Supplementary Table 4. SNPs significantly associated with postpartum depression in the meta-analysis of TMM-V2, TMM-NEO, and NGO-NEO cohorts considering the two most important confounding factors**

| **Associated SNPs** | | | | | **Meta-analysis** | | **TMM-V2** | | | | **TMM-NEO** | | | | **NGO-NEO** | | | |  | |
| --- | --- | --- | --- | --- | --- | --- | --- | --- | --- | --- | --- | --- | --- | --- | --- | --- | --- | --- | --- | --- |
| SNP ID | BP | Chr# | Locus | Gene | EA/RA | P | P | OR | LOG(OR)_SE | SE | P | OR | LOG(OR)_SE | SE | P | OR | LOG(OR)_SE | SE | Q statistic | I^2^ |
| rs377546683 | 58800275 | 1 | p32.2 | *DAB1* | C/G | 3.72E-09 | 0.08 | 1.07 | 0.06 | 0.81 | 3.85E-08 | 1.34 | 0.20 | 0.68 | 4.50E-03 | 0.64 | 0.60 | 1.34 | 0.25 | 0 |
| rs11940752 | 115442324 | 4 | q26 | *UGT8* | A/T | 1.36E-08 | 0.46 | 0.88 | 0.16 | 1.35 | 2.48E-08 | 2.12 | 0.14 | 0.18 | 4.35E-04 | 1.34 | 0.37 | 1.26 | 0.54 | 0 |
| rs141172317 | 169216010 | 5 | q35.1 | *DOCK2* | T/C | 1.66E-08 | 0.42 | 0.84 | 0.22 | 1.24 | 4.61E-08 | 2.78 | 0.19 | 0.18 | 4.62E-04 | 0.69 | 0.51 | 1.37 | 1.89 | 0 |
| rs117928019 | 169226605 | 5 | q35.1 | *DOCK2* | C/G | 1.49E-09 | 0.21 | 0.77 | 0.21 | 0.79 | 6.51E-09 | 2.69 | 0.17 | 0.17 | 6.50E-04 | 0.81 | 0.48 | 2.28 | 2.65 | 4.46 |
| rs76631412 | 169259673 | 5 | q35.1 | *DOCK2* | A/T | 1.99E-09 | 0.20 | 0.74 | 0.23 | 0.79 | 3.36E-08 | 2.71 | 0.18 | 0.18 | 3.19E-04 | 0.61 | 0.50 | 1.01 | 4.50 | 5.60 |
| rs118131805 | 169267720 | 5 | q35.1 | *DOCK2* | T/G | 1.99E-09 | 0.20 | 0.74 | 0.23 | 0.79 | 3.36E-08 | 2.71 | 0.18 | 0.18 | 3.19E-04 | 0.61 | 0.50 | 1.01 | 4.50 | 5.60 |
| rs188907279 | 125991737 | 8 | q24.13 | *ZNF572* | A/T | 4.08E-08 | 4.58E-08 | 1.47 | 0.17 | 0.45 | 0.54 | 1.17 | 0.21 | 1.32 | 6.01E-04 | 1.15 | 0.54 | 3.86 | 0.03 | 0 |
| rs504378 | 93336214 | 9 | q22.2 | *DIRAS2* | A/G | 4.12E-09 | 3.14E-06 | 1.25 | 0.09 | 0.39 | 2.59E-04 | 1.19 | 0.09 | 0.51 | 0.03 | 1.65 | 0.23 | 0.47 | 0.28 | 0 |
| rs690150 | 93359638 | 9 | q22.2 | *DIRAS2* | A/G | 2.84E-09 | 3.56E-06 | 1.25 | 0.09 | 0.39 | 4.85E-04 | 1.17 | 0.09 | 0.60 | 0.03 | 1.65 | 0.23 | 0.46 | 0.29 | 0 |
| rs491868 | 93349476 | 9 | q22.2 | *DIRAS2* | T/C | 4.24E-09 | 3.56E-06 | 1.26 | 0.09 | 0.38 | 5.29E-04 | 1.16 | 0.09 | 0.61 | 0.04 | 1.62 | 0.23 | 0.48 | 0.24 | 0 |
| rs689917 | 93359512 | 9 | q22.2 | *DIRAS2* | A/G | 3.96E-09 | 4.86E-06 | 1.25 | 0.09 | 0.39 | 5.13E-04 | 1.16 | 0.09 | 0.60 | 0.03 | 1.64 | 0.23 | 0.47 | 0.27 | 0 |
| rs474978 | 93352826 | 9 | q22.2 | *DIRAS2* | A/T | 2.99E-09 | 3.56E-06 | 1.25 | 0.09 | 0.39 | 5.13E-04 | 1.17 | 0.09 | 0.60 | 0.03 | 1.65 | 0.23 | 0.46 | 0.29 | 0 |
| rs690118 | 93356340 | 9 | q22.2 | *DIRAS2* | C/G | 2.93E-09 | 2.87E-06 | 1.24 | 0.09 | 0.40 | 3.98E-04 | 1.16 | 0.09 | 0.60 | 0.04 | 0.22 | 0.22 | 0.49 | 0.22 | 0 |
| rs690253 | 93352854 | 9 | q22.2 | *DIRAS2* | A/C | 1.46E-09 | 3.56E-06 | 1.25 | 0.09 | 0.39 | 4.61E-04 | 1.17 | 0.09 | 0.59 | 0.02 | 0.23 | 0.23 | 0.43 | 0.39 | 0 |
| rs1435984417 | 113929504 | 9 | q31.3 | *ZNF618* | C/G | 2.54E-08 | 0.002 | 1.30 | 0.19 | 0.72 | 1.22E-04 | 1.63 | 0.20 | 0.41 | 7.97E-03 | 0.47 | 0.47 | 0.57 | 0.42 | 0 |
| rs57705782 | 7451115 | 18 | p11.23 | *PTPRM* | T/C | 3.26E-09 | 3.98E-08 | 1.19 | 0.20 | 1.17 | 0.71 | 0.82 | 0.08 | 0.43 | 4.86E-03 | 0.87 | 0.87 | 0.56 | 4.24 | 9.84 |
| rs185293917 | 39604656 | 22 | q13.1 | *PDGFB* | G/C | 1.89E-09 | 0.64 | 1.12 | 0.24 | 2.16 | 1.52E-08 | 3.15 | 0.20 | 0.18 | 4.57E-03 | 0.55 | 0.55 | 1.33 | 0.52 | 0 |

The single nucleotide polymorphisms (SNPs) listed in the table were significantly associated with postpartum depression in the meta-analysis of Tohoku Medical Megabank Organization perinatal women sub-cohort genotyped by the Japonica Array version 2 (TMM-V2), Tohoku Medical Megabank Organization perinatal women sub-cohort genotyped by the Japonica Array version NEO (TMM-NEO), and Nagoya University perinatal women cohort genotyped by the Japonica Array version NEO (NGO-NEO) considering the two most important confounding factors. The I² values for the SNPs were zero or negligible (<25) in the meta-analysis of the TMM-V2, TMM-NEO, and NGO-NEO cohorts, indicating no or low heterogeneity among the three cohorts.

BP: base pair position, Chr #: chromosome number, EAF: effect allele frequency, OR: odds ratio, LOG(OR)_SE: standard error of the log odds ratio, SE: standard error

**Supplementary Table 5. The results of functional annotations**

| SNP | BP | Chr | Locus | Gene | CADD Phred Score |
| --- | --- | --- | --- | --- | --- |
| rs377546683 | 58800275 | 1 | p32.2 | *DAB1* | 16.5 |
| rs11940752 | 115442324 | 4 | q26 | *UGT8* | 9.1 |
| rs141172317 | 169216010 | 5 | q35.1 | *DOCK2* | 16.2 |
| rs117928019 | 169226605 | 5 | q35.1 | *DOCK2* | 16.2 |
| rs76631412 | 169259673 | 5 | q35.1 | *DOCK2* | 15.3 |
| rs118131805 | 169267720 | 5 | q35.1 | *DOCK2* | 15.4 |
| rs188907279 | 125991737 | 8 | q24.13 | *ZNF572* | 15.8 |
| rs504378 | 93336214 | 9 | q22.2 | *DIRAS2* | 19.8 |
| rs690150 | 93359638 | 9 | q22.2 | *DIRAS2* | 19.5 |
| rs491868 | 93349476 | 9 | q22.2 | *DIRAS2* | 19.9 |
| rs689917 | 93359512 | 9 | q22.2 | *DIRAS2* | 18.1 |
| rs474978 | 93352826 | 9 | q22.2 | *DIRAS2* | 18.5 |
| rs690118 | 93356340 | 9 | q22.2 | *DIRAS2* | 19.6 |
| rs690253 | 93352854 | 9 | q22.2 | *DIRAS2* | 19.9 |
| rs1435984417 | 113929504 | 9 | q31.3 | *ZNF618* | 10.9 |
| rs57705782 | 7451115 | 18 | p11.23 | *PTPRM* | 11.9 |
| rs185293917 | 39604656 | 22 | q13.1 | *PDGFB* | 12.4 |

TMM-V2: Tohoku Medical Megabank Organization perinatal women sub-cohort genotyped by the Japonica Array version 2, TMM-NEO: Tohoku Medical Megabank Organization perinatal women sub-cohort genotyped by the Japonica Array version NEO, NGO-NEO: Nagoya University perinatal women cohort genotyped by the Japonica Array version NEO, CADD Phred Score: Combined Annotation-Dependent Depletion Phred Score

**Supplementary Table 6. The top 46 significant pathways from the pathway analyses**

| Gene Set | Description | Ratio | P Value |
| --- | --- | --- | --- |
| hsa04730 | Long-term depression | 2.1348 | 1.02E-05 |
| hsa00410 | beta-Alanine metabolism | 1.9947 | 4.50E-03 |
| hsa04970 | Salivary secretion | 1.9631 | 3.32E-06 |
| hsa05412 | Arrhythmogenic right ventricular cardiomyopathy (ARVC) | 1.9631 | 3.13E-05 |
| hsa04912 | GnRH signaling pathway | 1.8997 | 8.87E-06 |
| hsa04664 | Fc epsilon RI signaling pathway | 1.8837 | 1.78E-04 |
| hsa04012 | ErbB signaling pathway | 1.8707 | 3.69E-05 |
| hsa01521 | EGFR tyrosine kinase inhibitor resistance | 1.845 | 1.07E-04 |
| hsa04916 | Melanogenesis | 1.8367 | 1.47E-05 |
| hsa04724 | Glutamatergic synapse | 1.821 | 6.12E-06 |
| hsa04750 | Inflammatory mediator regulation of TRP channels | 1.7846 | 5.15E-05 |
| hsa04921 | Oxytocin signaling pathway | 1.7726 | 7.98E-07 |
| hsa04911 | Insulin secretion | 1.7668 | 2.32E-04 |
| hsa05226 | Gastric cancer | 1.749 | 2.03E-06 |
| hsa04514 | Cell adhesion molecules (CAMs) | 1.7484 | 3.06E-06 |
| hsa05231 | Choline metabolism in cancer | 1.74 | 1.22E-04 |
| hsa04974 | Protein digestion and absorption | 1.7177 | 3.58E-04 |
| hsa04540 | Gap junction | 1.7065 | 4.98E-04 |
| hsa04925 | Aldosterone synthesis and secretion | 1.7024 | 3.03E-04 |
| hsa04310 | Wnt signaling pathway | 1.6942 | 1.14E-05 |
| hsa05214 | Glioma | 1.6797 | 2.39E-03 |
| hsa04390 | Hippo signaling pathway | 1.6635 | 1.53E-05 |
| hsa04261 | Adrenergic signaling in cardiomyocytes | 1.6563 | 3.43E-05 |
| hsa04725 | Cholinergic synapse | 1.6563 | 2.47E-04 |
| hsa05205 | Proteoglycans in cancer | 1.6481 | 1.35E-06 |
| hsa04270 | Vascular smooth muscle contraction | 1.6427 | 1.88E-04 |
| hsa05225 | Hepatocellular carcinoma | 1.6301 | 1.65E-05 |
| hsa04015 | Rap1 signaling pathway | 1.6295 | 1.91E-06 |
| hsa04360 | Axon guidance | 1.6153 | 1.69E-05 |
| hsa04934 | Cushing syndrome | 1.6062 | 6.74E-05 |
| hsa04072 | Phospholipase D signaling pathway | 1.6034 | 1.10E-04 |
| hsa04022 | cGMP-PKG signaling pathway | 1.5988 | 5.06E-05 |
| hsa04926 | Relaxin signaling pathway | 1.5969 | 2.95E-04 |
| hsa04020 | Calcium signaling pathway | 1.593 | 2.11E-05 |
| hsa05224 | Breast cancer | 1.5624 | 2.69E-04 |
| hsa04727 | GABAergic synapse | 1.556 | 4.70E-03 |
| hsa05410 | Hypertrophic cardiomyopathy (HCM) | 1.5433 | 6.98E-03 |
| hsa04211 | Longevity regulating pathway | 1.5385 | 5.68E-03 |
| hsa04150 | mTOR signaling pathway | 1.5211 | 5.62E-04 |
| hsa04510 | Focal adhesion | 1.5093 | 1.12E-04 |
| hsa04530 | Tight junction | 1.5069 | 3.65E-04 |
| hsa04010 | MAPK signaling pathway | 1.4523 | 2.51E-05 |
| hsa04014 | Ras signaling pathway | 1.4279 | 3.68E-04 |
| hsa04810 | Regulation of actin cytoskeleton | 1.4101 | 9.92E-04 |
| hsa05200 | Pathways in cancer | 1.3435 | 1.13E-05 |
| hsa04080 | Neuroactive ligand-receptor interaction | 1.2756 | 8.16E-03 |

*FDR <0.05

**Supplementary Table 7. Previous GWAS studies**

| **Author(s)** | **Year** | **Sample size** | **Genome-wide significant loci** | **Overlaps with the present study** | **Disease** |
| --- | --- | --- | --- | --- | --- |
| Glessner ^36^ | 2010 | 1,693 cases, 4,506 controls | *SLIT3, CCDC99, DOCK2* | *DOCK2* | MDD |
| Sullivan ^59^ | 2013 | 9,240 cases, 9,519 controls | No SNPs | / | MDD |
| N Davies ^60^ | 2014 | 50 monozygotic twin pairs | *CADPS1, PTPRM, ZBTB20* | *PTPRM* | MDD |
| CONVERGE Consortium ^61^ | 2015 | 5,303 cases, 5,337 controls | *SIRT1 and LHPP* gene loci (Chr 10) | / | MDD |
| Chen ^62^ | 2017 | 56,569 SNPs, SCZ119, BD253, MDD177 | *DOCK2, CDH6, CDH9, CDH10, CDH12* | *DOCK2* | SCZ, BD, MDD |
| Howard ^63^ | 2019 | 246,363 cases, 561,190 controls | 87 associated variants | / | Depression |
| Hüls ^29^ | 2020 | 608 participants | *YOD1, UGT8, FNDC3B, SLIT2* | *UGT8* | MDD |
| Giannakopoulou ^64^ | 2021 | 15,771 cases, 178,777 controls | rs4656484  rs10240457 | / | Depression |
| Thalamuthu ^65^ | 2022 | 3,510 cases, 6,057 controls | 48 SNPs | / | MDD |
| Qi ^16^ | 2022 | 503,325 participants | *DAB1, ROBO2, CACNA1C, GRK5* | *DAB1* | BD, depression |
| Lee ^66^ | 2010 | 1,000 cases, 1,000 controls | No SNPs | / | BD |
| Reif ^43^ | 2011 | 600 ADHD, 420 controls | *DIRAS2* | *DIRAS2* | BD, ADHD |
| Ikeda ^67^ | 2017 | 2,964 cases, 61,887 controls | 11q12.2, *FADS1/2/3* | / | BD |
| Li ^68^ | 2021 | 1,822 cases, 4,650 controls | *TMEM108, VRK2, RHEBL1* | / | BD |
| Mullins ^69^ | 2021 | 41,917 cases, 371,549 controls | 64 associated genomic loci | *DOCK2* | BD |

This table summarizes previous studies, including the first author, year of publication, sample size, results, and diseases, based on GWAS analysis.

**References**

1. Chang CC, Chow CC, Tellier LC, Vattikuti S, Purcell SM, Lee JJ. Second-generation PLINK: rising to the challenge of larger and richer datasets. *Gigascience.* 2015; **4**: 7.

2. Jiang L, Zheng Z, Qi T et al. A resource-efficient tool for mixed model association analysis of large-scale data. *Nat Genet.* 2019; **51**: 1749-1755.

3. Yang J, Lee SH, Goddard ME, Visscher PM. GCTA: a tool for genome-wide complex trait analysis. *Am J Hum Genet.* 2011; **88**: 76-82.

4. Jiang L, Zheng Z, Fang H, Yang J. A generalized linear mixed model association tool for biobank-scale data. *Nat Genet.* 2021; **53**: 1616-1621.

5. Willer CJ, Li Y, Abecasis GR. METAL: fast and efficient meta-analysis of genomewide association scans. *Bioinformatics.* 2010; **26**: 2190-1.

6. Qiu F, Tang R, Zuo X et al. A genome-wide association study identifies six novel risk loci for primary biliary cholangitis. *Nat Commun.* 2017; **8**: 14828.

7. El-Husseini ZW, Gosens R, Dekker F, Koppelman GH. The genetics of asthma and the promise of genomics-guided drug target discovery. *Lancet Respir Med.* 2020; **8**: 1045-1056.

8. Barton AR, Sherman MA, Mukamel RE, Loh PR. Whole-exome imputation within UK Biobank powers rare coding variant association and fine-mapping analyses. *Nat Genet.* 2021; **53**: 1260-1269.

9. Huffman JE, Butler-Laporte G, Khan A et al. Multi-ancestry fine mapping implicates OAS1 splicing in risk of severe COVID-19. *Nat Genet.* 2022; **54**: 125-127.

10. Hutchinson A, Asimit J, Wallace C. Fine-mapping genetic associations. *Hum Mol Genet.* 2020; **29**: R81-r88.

11. Kimbrel NA, Ashley-Koch AE, Qin XJ et al. A genome-wide association study of suicide attempts in the million veterans program identifies evidence of pan-ancestry and ancestry-specific risk loci. *Mol Psychiatry.* 2022; **27**: 2264-2272.

12. Lundberg SM, Lee S-I. A unified approach to interpreting model predictions. *Advances in neural information processing systems.* 2017; **30**.

13. Tsuneura Y, Nakai T, Mizoguchi H, Yamada K. New Strategies for the Treatment of Neuropsychiatric Disorders Based on Reelin Dysfunction. *Int J Mol Sci.* 2022; **23**.

14. Knable MB, Barci BM, Webster MJ, Meador-Woodruff J, Torrey EF. Molecular abnormalities of the hippocampus in severe psychiatric illness: postmortem findings from the Stanley Neuropathology Consortium. *Mol Psychiatry.* 2004; **9**: 609-20, 544.

15. Fatemi SH, Earle JA, McMenomy T. Reduction in Reelin immunoreactivity in hippocampus of subjects with schizophrenia, bipolar disorder and major depression. *Mol Psychiatry.* 2000; **5**: 654-63, 571.

16. Qi X, Jia Y, Pan C et al. Index of multiple deprivation contributed to common psychiatric disorders: A systematic review and comprehensive analysis. *Neurosci Biobehav Rev.* 2022; **140**: 104806.

17. Goes FS, Willour VL, Zandi PP et al. Sex-specific association of the Reelin gene with bipolar disorder. *Am J Med Genet B Neuropsychiatr Genet.* 2010; **153b**: 549-553.

18. Asor E, Ben-Shachar D. Gene expression dynamics following mithramycin treatment: A possible model for post-chemotherapy cognitive impairment. *Clin Exp Pharmacol Physiol.* 2018; **45**: 1028-1037.

19. Gao H, Tao Y, He Q, Song F, Saffen D. Functional enrichment analysis of three Alzheimer's disease genome-wide association studies identities DAB1 as a novel candidate liability/protective gene. *Biochem Biophys Res Commun.* 2015; **463**: 490-5.

20. Sánchez-Sánchez SM, Magdalon J, Griesi-Oliveira K et al. Rare RELN variants affect Reelin-DAB1 signal transduction in autism spectrum disorder. *Hum Mutat.* 2018; **39**: 1372-1383.

21. Guidotti A, Grayson DR, Caruncho HJ. Epigenetic RELN Dysfunction in Schizophrenia and Related Neuropsychiatric Disorders. *Front Cell Neurosci.* 2016; **10**: 89.

22. Imai H, Shoji H, Ogata M et al. Dorsal Forebrain-Specific Deficiency of Reelin-Dab1 Signal Causes Behavioral Abnormalities Related to Psychiatric Disorders. *Cereb Cortex.* 2017; **27**: 3485-3501.

23. Lammert DB, Howell BW. RELN Mutations in Autism Spectrum Disorder. *Front Cell Neurosci.* 2016; **10**: 84.

24. Fatemi SH, Snow AV, Stary JM et al. Reelin signaling is impaired in autism. *Biol Psychiatry.* 2005; **57**: 777-87.

25. Li J, Liu J, Zhao L et al. Association study between genes in Reelin signaling pathway and autism identifies DAB1 as a susceptibility gene in a Chinese Han population. *Prog Neuropsychopharmacol Biol Psychiatry.* 2013; **44**: 226-32.

26. Shen Y, Xun G, Guo H et al. Association and gene-gene interactions study of reelin signaling pathway related genes with autism in the Han Chinese population. *Autism Res.* 2016; **9**: 436-42.

27. Teixeira CM, Masachs N, Muhaisen A et al. Transient downregulation of Dab1 protein levels during development leads to behavioral and structural deficits: relevance for psychiatric disorders. *Neuropsychopharmacology.* 2014; **39**: 556-68.

28. Aston C, Jiang L, Sokolov BP. Transcriptional profiling reveals evidence for signaling and oligodendroglial abnormalities in the temporal cortex from patients with major depressive disorder. *Mol Psychiatry.* 2005; **10**: 309-22.

29. Hüls A, Robins C, Conneely KN et al. Association between DNA methylation levels in brain tissue and late-life depression in community-based participants. *Transl Psychiatry.* 2020; **10**: 262.

30. Le-Niculescu H, Kurian SM, Yehyawi N et al. Identifying blood biomarkers for mood disorders using convergent functional genomics. *Mol Psychiatry.* 2009; **14**: 156-74.

31. Malhotra D, McCarthy S, Michaelson JJ et al. High frequencies of de novo CNVs in bipolar disorder and schizophrenia. *Neuron.* 2011; **72**: 951-63.

32. Narayan S, Head SR, Gilmartin TJ, Dean B, Thomas EA. Evidence for disruption of sphingolipid metabolism in schizophrenia. *J Neurosci Res.* 2009; **87**: 278-88.

33. Davis KL, Stewart DG, Friedman JI et al. White matter changes in schizophrenia: evidence for myelin-related dysfunction. *Arch Gen Psychiatry.* 2003; **60**: 443-56.

34. Kubicki M, McCarley RW, Shenton ME. Evidence for white matter abnormalities in schizophrenia. *Curr Opin Psychiatry.* 2005; **18**: 121-34.

35. Ji L, Xu S, Luo H, Zeng F. Insights from DOCK2 in cell function and pathophysiology. *Front Mol Biosci.* 2022; **9**: 997659.

36. Glessner JT, Wang K, Sleiman PM et al. Duplication of the SLIT3 locus on 5q35.1 predisposes to major depressive disorder. *PLoS One.* 2010; **5**: e15463.

37. Perez JM, Berto S, Gleason K et al. Hippocampal subfield transcriptome analysis in schizophrenia psychosis. *Mol Psychiatry.* 2021; **26**: 2577-2589.

38. Jensen PS. Inattention and impulsivity in children and adolescents: a developmental and contextual framework to understand attention-deficit hyperactivity disorder and its variants. 2021.

39. Bainomugisa CK, Sutherland HG, Parker R et al. Using Monozygotic Twins to Dissect Common Genes in Posttraumatic Stress Disorder and Migraine. *Front Neurosci.* 2021; **15**: 678350.

40. Mehta D, Bruenig D, Carrillo-Roa T et al. Genomewide DNA methylation analysis in combat veterans reveals a novel locus for PTSD. *Acta Psychiatr Scand.* 2017; **136**: 493-505.

41. Mehta D, Pelzer ES, Bruenig D et al. DNA methylation from germline cells in veterans with PTSD. *J Psychiatr Res.* 2019; **116**: 42-50.

42. Kim MA, Lee EJ, Yang W, Shin HY, Kim YH, Kim JH. Identification of a novel gene signature in second-trimester amniotic fluid for the prediction of preterm birth. *Sci Rep.* 2022; **12**: 3085.

43. Reif A, Nguyen TT, Weissflog L et al. DIRAS2 is associated with adult ADHD, related traits, and co-morbid disorders. *Neuropsychopharmacology.* 2011; **36**: 2318-27.

44. Grünewald L, Landaas ET, Geissler J et al. Functional Impact of An ADHD-Associated DIRAS2 Promoter Polymorphism. *Neuropsychopharmacology.* 2016; **41**: 3025-3031.

45. Demontis D, Walters RK, Martin J et al. Discovery of the first genome-wide significant risk loci for attention deficit/hyperactivity disorder. *Nat Genet.* 2019; **51**: 63-75.

46. Grünewald L, Chiocchetti AG, Weber H et al. Knockdown of the ADHD Candidate Gene Diras2 in Murine Hippocampal Primary Cells. *J Atten Disord.* 2021; **25**: 572-583.

47. Grünewald L, Becker N, Camphausen A et al. Expression of the ADHD candidate gene Diras2 in the brain. *J Neural Transm (Vienna).* 2018; **125**: 913-923.

48. Detera-Wadleigh SD, Badner JA, Berrettini WH et al. A high-density genome scan detects evidence for a bipolar-disorder susceptibility locus on 13q32 and other potential loci on 1q32 and 18p11.2. *Proc Natl Acad Sci U S A.* 1999; **96**: 5604-9.

49. Drazinic CM, Ercan-Sencicek AG, Gault LM et al. Rapid array-based genomic characterization of a subtle structural abnormality: a patient with psychosis and der(18)t(5;18)(p14.1;p11.23). *Am J Med Genet A.* 2005; **134**: 282-9.

50. Washizuka S, Kakiuchi C, Mori K et al. Association of mitochondrial complex I subunit gene NDUFV2 at 18p11 with bipolar disorder. *Am J Med Genet B Neuropsychiatr Genet.* 2003; **120b**: 72-8.

51. Walsh T, McClellan JM, McCarthy SE et al. Rare structural variants disrupt multiple genes in neurodevelopmental pathways in schizophrenia. *Science.* 2008; **320**: 539-43.

52. Vawter MP, Crook JM, Hyde TM et al. Microarray analysis of gene expression in the prefrontal cortex in schizophrenia: a preliminary study. *Schizophr Res.* 2002; **58**: 11-20.

53. Condra JA, Neibergs H, Wei W, Brennan MD. Evidence for two schizophrenia susceptibility genes on chromosome 22q13. *Psychiatr Genet.* 2007; **17**: 292-8.

54. McGuffin P, Tandon K, Corsico A. Linkage and association studies of schizophrenia. *Curr Psychiatry Rep.* 2003; **5**: 121-7.

55. Takahashi S, Cui YH, Kojima T et al. Family-based association study of markers on chromosome 22 in schizophrenia using African-American, European-American, and Chinese families. *Am J Med Genet B Neuropsychiatr Genet.* 2003; **120b**: 11-7.

56. Zai G, Zai C, Tiwari A et al. Weak association of the platelet-derived growth factor beta (PDGFB) and PDGF receptor beta (PDGFRB) genes with schizophrenia and schizoaffective disorder. *World J Biol Psychiatry.* 2011; **12**: 127-33.

57. Hayashi T, Legati A, Nishikawa T, Coppola G. First Japanese family with primary familial brain calcification due to a mutation in the PDGFB gene: an exome analysis study. *Psychiatry Clin Neurosci.* 2015; **69**: 77-83.

58. Lee SA, Huang KC. Epigenetic profiling of human brain differential DNA methylation networks in schizophrenia. *BMC Med Genomics.* 2016; **9**: 68.

59. Ripke S, Wray NR, Lewis CM et al. A mega-analysis of genome-wide association studies for major depressive disorder. *Mol Psychiatry.* 2013; **18**: 497-511.

60. Davies MN, Krause L, Bell JT et al. Hypermethylation in the ZBTB20 gene is associated with major depressive disorder. *Genome Biol.* 2014; **15**: R56.

61. Sparse whole-genome sequencing identifies two loci for major depressive disorder. *Nature.* 2015; **523**: 588-91.

62. Chen X, Long F, Cai B, Chen X, Chen G. A novel relationship for schizophrenia, bipolar and major depressive disorder Part 5: a hint from chromosome 5 high density association screen. *Am J Transl Res.* 2017; **9**: 2473-2491.

63. Howard DM, Adams MJ, Clarke TK et al. Genome-wide meta-analysis of depression identifies 102 independent variants and highlights the importance of the prefrontal brain regions. *Nat Neurosci.* 2019; **22**: 343-352.

64. Giannakopoulou O, Lin K, Meng X et al. The Genetic Architecture of Depression in Individuals of East Asian Ancestry: A Genome-Wide Association Study. *JAMA Psychiatry.* 2021; **78**: 1258-1269.

65. Thalamuthu A, Mills NT, Berger K et al. Genome-wide interaction study with major depression identifies novel variants associated with cognitive function. *Mol Psychiatry.* 2022; **27**: 1111-1119.

66. Lee MT, Chen CH, Lee CS et al. Genome-wide association study of bipolar I disorder in the Han Chinese population. *Mol Psychiatry.* 2011; **16**: 548-56.

67. Ikeda M, Takahashi A, Kamatani Y et al. A genome-wide association study identifies two novel susceptibility loci and trans population polygenicity associated with bipolar disorder. *Mol Psychiatry.* 2018; **23**: 639-647.

68. Li HJ, Zhang C, Hui L et al. Novel Risk Loci Associated With Genetic Risk for Bipolar Disorder Among Han Chinese Individuals: A Genome-Wide Association Study and Meta-analysis. *JAMA Psychiatry.* 2021; **78**: 320-330.

69. Mullins N, Forstner AJ, O'Connell KS et al. Genome-wide association study of more than 40,000 bipolar disorder cases provides new insights into the underlying biology. *Nat Genet.* 2021; **53**: 817-829.
